# Hochuekkito accelerates recovery from cisplatin induced-muscle atrophy accompanied by slow-twitch fiber-specific microRNA upregulation in mice

**DOI:** 10.3389/fphar.2025.1502563

**Published:** 2025-05-15

**Authors:** Hitomi Sekine, Chinami Matsumoto, Naoki Fujitsuka, Sachiko Mogami, Shunsuke Ohnishi, Hiroshi Takeda

**Affiliations:** ^1^ TSUMURA Kampo Research Laboratories, Research and Development Division, TSUMURA & CO., Ibaraki, Japan; ^2^ Faculty of Pharmaceutical Sciences, Hokkaido University, Sapporo, Hokkaido, Japan; ^3^ Gastroenterology, Caress Memorial Hospital, Sapporo, Hokkaido, Japan

**Keywords:** cisplatin, muscle atrophy, hochuekkito, myogenesis, differentiation, regeneration, slow-twitch fibers, microRNA

## Abstract

**Introduction:**

Cis-diamine-dichloro-platinum (II) (cisplatin) is a widely prescribed anticancer drug known to induce severe side effects, including skeletal muscle atrophy. As muscle atrophy negatively impacts the prognosis and survival of cancer patients, elucidation of its pathogenesis and development of preventive and treatment methods is important. Hochuekkito (HET), a Japanese Kampo medicine, has been reported to improve decreased physical activity and muscle weight in various animal models, but not in cisplatin-induced muscle atrophy. Therefore, this study aimed to clarify the characteristics of cisplatin-induced muscle atrophy and the therapeutic effects of HET, especially with emphasis in the recovery phase.

**Method:**

Eight-week-old male C57BL/6J mice were administered with cisplatin (3 mg/kg/day, intraperitoneally) from Day0 to Day3 and provided HET-containing food from 2 weeks before cisplatin administration until Day14. Muscle weight and performance were evaluated and the underlying mechanisms were investigated by using gene expression, immunohistochemistry, and microRNA (miRNA)-sequence analyses.

**Results:**

Cisplatin administration continued to reduce gastrocnemius muscle weight until Day14, even after recovery from transient decrease in food intake and body weight and was accompanied by decreased locomotor activity and grip strength, presumably due to the continuous suppression of the mRNA expression of *Myogenic differentiation 1*. Although HET did not suppress the activation of muscle protein degradation or increase myogenic regulatory factor expression, it restored decreased locomotor activity and gastrocnemius muscle weight by inducing an increase in the weight of the red muscle region, which contains slow-twitch fibers. Immunohistochemical analysis showed that red muscle slow-twitch fiber cross-sectional area was increased by HET treatment. The levels of miRNAs involved in muscle atrophy and aging, such as miR-29a/b and miR-34a, were increased by cisplatin; conversely, HET increased the expression of miR-1a-1 and miR-1a-2, which reportedly enhance muscle differentiation, and miR-206, which enhances slow muscle fiber differentiation and thickening.

**Conclusion:**

HET increased locomotor activity and muscle weight in cisplatin-induced muscle atrophy model mice, probably by enhancing myogenesis in slow-twitch fibers, which was related to miRNA expression changes. Thus, HET may be useful in treating cisplatin-induced muscle atrophy.

## 1 Introduction

Cis-diamine-dichloro-platinum (II) (cisplatin, CDDP) is a widely prescribed cancer drug, particularly for patients with bladder, head and neck, lung, ovarian, and testicular cancers (Dasari and Tchounwou, 2014). Cisplatin has several severe side effects such as myelosuppression, gastrointestinal toxicity, nephrotoxicity, ototoxicity, and neurotoxicity (Pabla and Dong, 2008; Santos et al., 2020). Recently, cisplatin-induced skeletal muscle atrophy has emerged as an important clinical issue, as it negatively affects the outcomes of cancer treatment (Daly et al., 2018; Bozzetti, 2020; Lin et al., 2021). Accumulating evidence shows that cisplatin induces skeletal muscle atrophy by both reducing protein synthesis and activating protein degrading systems such as those involving the muscle-specific ubiquitin ligases atrogin-1 and muscle RING-finger 1 (MuRF1) (Sakai et al., 2014; Lala-Tabbert et al., 2019; Conte et al., 2020; Zhang et al., 2020; Huang K. C. et al., 2023); additionally, mitochondrial dysfunction and reactive oxygen species generation have also been suggested to be involved in this process (Sirago et al., 2017; Seo et al., 2021; Matsumoto et al., 2022). Cisplatin is generally administered intermittently every few weeks, and given that skeletal muscles have high plasticity and regenerative capacity (Mukund and Subramaniam, 2020), skeletal muscle damage caused by it should recover to some extent during the drug-free period. However, most studies to date have focused on the acute induction phase after cisplatin administration, and information on the recovery process is lacking.

Many effective preventive and therapeutic methods for cisplatin-induced muscle atrophy have been evaluated in preclinical studies (Daly et al., 2018; Davis and Panikkar, 2019; Yoshizawa et al., 2019; Bozzetti, 2020; Lin et al., 2021; Ikeno et al., 2022; Huang K. C. et al., 2023); however, their clinical efficacy in cancer patients has not yet been proven. Kampo medicines (traditional Japanese medicines), can be useful in alleviating subjective symptoms such as fatigue in patients with cancer and in reducing several side effects of cancer chemotherapy (Zhang et al., 2021; Motoo and Cameron, 2022). Of these, hochuekkito (HET) is particularly noteworthy, as it is reported to be effective in treating cachexia in patients with genitourinary cancer and chronic fatigue syndrome in clinical settings (Kuchta and Cameron, 2020) and skeletal muscle atrophy in several mouse models, including cancer-associated cachexia induced by implanting colon 26 adenocarcinoma cells (Yae et al., 2012), weightlessness-induced muscle atrophy (Zhu et al., 2017; Yakabe et al., 2022) and amyotrophic lateral sclerosis (Cai and Yang, 2019). However, the effects of HET on cisplatin-induced muscle atrophy have not yet been investigated.

Micro RNAs (miRNAs) are 19–25 nucleotide non-coding RNA molecules that are known to modulate the expression of numerous genes at both the transcriptional and post-transcriptional levels and to exhibit tissue-specific and developmental expression patterns (Lu and Rothenberg, 2018; Seyhan, 2024). Several miRNAs play fundamental roles in skeletal muscle development, differentiation, atrophy, regeneration, hypertrophy, and functioning (Sun et al., 2022; Jung et al., 2024). The role of miRNAs in the pathogenesis of skeletal muscle atrophy has been extensively investigated, and several candidate miRNAs that are directly involved in skeletal muscle atrophy have been identified (Yang et al., 2020; Cannataro et al., 2021). However, there have been no reports yet on the involvement of skeletal muscle microRNAs in cisplatin-induced muscle atrophy. Furthermore, the relationship between pharmacological effects of HET and miRNAs has not been studied.

In this study, we first examined the decrease in skeletal muscle weights and muscle performance after 4 days-cisplatin administration to investigate its effect over time, including the recovery phase till Day14. Secondary, we investigated the effect of HET on cisplatin-induced skeletal muscle atrophy by focusing specifically on the recovery phase, Day14. Furthermore, to clarify the mechanism of HET, we comprehensively examined the changes in skeletal muscle miRNA expression.

## 2 Materials and methods

### 2.1 Animals

Male C57BL/6J mice (8-week old at the time of cisplatin administration) were purchased from Jackson Laboratory Japan, Inc. (Kanagawa, Japan). All animals were housed separately under environmentally controlled ambient temperature (23°C ± 3°C), humidity (50% ± 20%), and lighting (12 h light/dark cycle) conditions and had *ad libitum* access to water and food.

This study was approved by the Experimental Animal Ethics Committee of TSUMURA & CO. (Tokyo, Japan; permit no. 18-043, 19-013, 23-005), and all experimental procedures were performed in accordance with the ARRIVE guidelines and the guidelines of the National Institutes of Health for the Care and Use of Laboratory Animals.

### 2.2 Test substances

Cisplatin was purchased from Fujifilm Wako (Osaka, Japan) and dissolved in saline at a concentration of 3 mg/10 mL. HET was manufactured by TSUMURA & CO. (Tokyo, Japan) as the intermediate product (extract powder, Lot No. 2240041020, 2200041020, 2210041010, 2230041010) of ‘TSUMURA Hochuekkito Extract Granules for Ethical Use’ as shown in their website (https://www.tsumura.co.jp/english/kampo/, and https://www.tsumura.co.jp/english/ir/library/integrated-report/). The extract quality was standardized based on good manufacturing practices, as defined by the Ministry of Health, Labour, and Welfare of Japan and are in accordance with the Japanese Pharmacopoeia (JP) (https://www.pmda.go.jp/english/rs-sb-std/standards-development/jp/0029.html, https://www.mhlw.go.jp/content/11120000/000904450.pdf). Briefly, it was manufactured by spray-drying the hot-water extract of the following 10 crude drugs: 4.0 parts JP Astragalus root [*Astragalus membranaceus* Bunge, or *Astragalus mongholicus* Bunge (Leguminosae), radix], 4.0 parts JP Atractylodes lancea rhizome [*Atractylodes lancea* De Candolle, or *Atractylodes schinensis* Koidzumi (Asteraceae), rhizome], 4.0 parts JP Ginseng [*Panax ginseng* C. A. Meyer (*Panax schinseng* Nees) (Araliaceae), radix], 3.0 parts JP Japanese angelica root [*Angelica acutiloba* Kitagawa, or *Angelica acutiloba* Kitagawa var. *sugiyamae* Hikino (Umbelliferae), radix], 2.0 parts JP Bupleurum root [*Bupleurum falcatum* Linné (Umbelliferae), radix], 2.0 parts JP Jujube [*Ziziphus jujuba* Miller var. *inermis* Rehder (Rhamnaceae), fructus], 2.0 parts JP Citrus unshiu peel [*Citrus unshiu* Marcowicz, or *Citrus reticulata* Blanco (Rutaceae), pericarpium], 1.5 parts JP Glycyrrhiza [*Glycyrrhiza uralensis* Fischer, or *Glycyrrhiza glabra* Linné (Leguminosae), radix], 1.0 parts JP Cimicifuga rhizome [*Cimicifuga simplex* Turczaninow, *Cimicifuga dahurica* Maximowicz, *Cimicifuga foetida* Linné, or *Cimicifuga heracleifolia* Komarov (Ranunculaceae), rhizome], and 0.5 parts JP ginger [*Zingiber officinale* Roscoe (Zingiberaceae), rhizome]. The plants were identified by their external morphology and marker metabolites as per the JP and company standards. A three-dimensional high-performance liquid chromatogram of HET is presented in Supplementary Figure S1. HET was administered to mice by mixing it into standard chow (MF; Oriental Yeast Co., Ltd., Tokyo, Japan) at a concentration of 1.5% or 3%, which are approximately 2 and 4 times the human equivalent dose, respectively. The doses were determined according to previous reports (Nahata et al., 2021; Nahata et al., 2022; Okugawa et al., 2024).

### 2.3 Experimental protocol

#### 2.3.1 Cisplatin-induced muscle atrophy model

**FIGURE 1:**
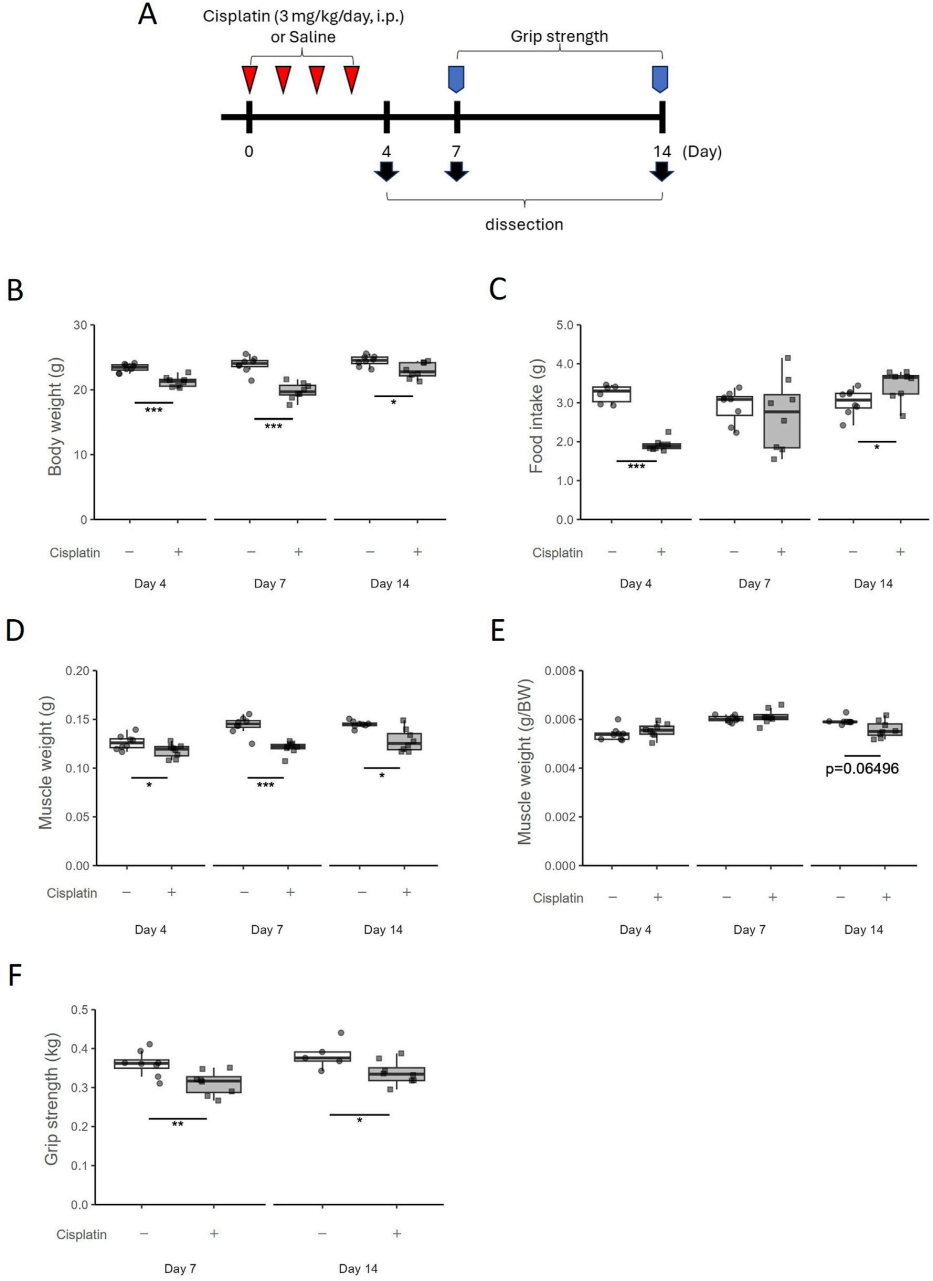
Temporal changes in the cisplatin-induced muscle atrophy model. **(A)** Experimental design. **(B)** Body weight was measured on Day4, Day7, and Day14. *n* = 8 for each group. **(C)** Food intake was measured on Day4, Day7, and Day14. *n* = 6 in the vehicle group on Day4; *n* = 8 for the cisplatin group on Day4 and the vehicle and cisplatin groups on Day7 and Day14. **(D)** Hindlimb skeletal muscle (gastrocnemius and plantaris muscle) weight and **(E)** muscle weights per gram body weight were measured on Day4, Day7, and Day14. *n* = 8 for each group. **(F)** Grip strength of limb muscles was measured on Day7 and Day14. Grip strength was obtained by averaging five measurements. *n* = 8 for the vehicle group on Day7 and the cisplatin group on Day7 and Day14; *n* = 5 for the vehicle group on Day14. Data are presented as Tukey’s box plots. The white box and round dots and the gray box and square dots represent the vehicle and cisplatin groups, respectively. The dots represent the values for each animal. BW; body weight. *p < 0.05, **p < 0.01, ***p < 0.001 vs vehicle (Student’s or Aspin–Welch *t*-test or Wilcoxon rank-sum test).

Animals were randomly assigned two groups (*n* = 8 in each group). The cisplatin group was intraperitoneally administered cisplatin to mice (3 mg/kg/day) for 4 days to induce muscle atrophy. The vehicle group was intraperitoneally administered saline (10 mL/kg/day). The first day of cisplatin administration was defined as Day0, and the mice were sacrificed on Day4, 7, or 14 (Figure 1A).

#### 2.3.2 Effects of HET on cisplatin-induced muscle atrophy model mice

Animals were randomly assigned three or four groups (*n* = 10 or 15 in each group); vehicle group (saline + MF diet), cisplatin group (cisplatin + MF diet), HET 1.5% group (cisplatin +1.5% HET-containing MF) and HET 3% group (cisplatin +3% HET-containing MF). Animals were provided with HET-containing MF solid food from 2 weeks before administering cisplatin to habituate them to the specialty feed, as it differs in texture and stiffness compared to the standard chow and to avoid the simultaneous effect of cisplatin which decreases food intake, until the day of dissection. The vehicle group was provided with a similarly manufactured MF diet without HET (Oriental Yeast Co., Ltd., Tokyo, Japan) (Figure 3A).

Body weight was measured daily after administration. Food intake and locomotor activity were measured until Day13. The Day0 values represent those obtained from day −1 to day 0. Locomotor activity was assessed using an infrared ray sensor (NS-AS02; Neuroscience, Inc., Tokyo, Japan). The NS-DAS-16NS system and Act-1 Light software (Neuroscience, Inc., Tokyo, Japan) were used for data collection and analysis. The locomotor activity between 9:00 a.m. and 12:00 a.m. was excluded from the analysis as this period was when researchers were working beside the sensor. A grip strength meter for mice (MK-380M; Muromachi Kikai Co., Ltd., Tokyo, Japan) was used to measure the limb muscle grip strength of the mice on Day7, 13, or 14. Grip strength was calculated as the average of five measurements per mouse.

The animals were anesthetized using isoflurane or sevoflurane before sacrifice. The gastrocnemius muscles, including the plantar muscles, were collected and used for gene expression analysis or fixed in formaldehyde and used for immunohistochemical staining. Additionally, the white and red regions of the gastrocnemius muscle were visually separated according to their color, as previously described (Dimauro et al., 2019; Halle et al., 2022) before being weighed, frozen in liquid nitrogen, and stored at −80°C until further use.

### 2.4 Gene expression analysis

Total RNA was extracted from muscle samples using the RNeasy Mini Kit (74106; Qiagen, Valencia, CA, United States) and reverse transcribed to cDNA using the TaqMan High-Capacity cDNA Reverse Transcription Kit (4374967; Applied Biosystems, Waltham, MA, United States). DNA was extracted from muscle samples using the QIAamp DNA Mini Kit (51304; Qiagen, Valencia, CA, United States). mRNA expression and mitochondrial DNA (mtDNA) copy number was analyzed using quantitative real-time polymerase chain reaction (4444557; TaqMan Fast Advanced Master Mix, Applied Biosystems, Waltham, MA, United States) using TaqMan gene-specific primers (Thermo Fisher Scientific, Waltham, MA, United States) on a Quant Studio 7 Flex Real-Time PCR System (Applied Biosystems, Waltham, MA, United States). mRNA expression was normalized to that of *glyceraldehyde 3-phosphate dehydrogenase* (*Gapdh*), and the mtDNA copy number was normalized using the nuclear gene *ribonuclease P RNA component H1* (*Rpph1*). The TaqMan gene-specific primers used for the experiments are provided in the Supplementary File.

### 2.5 miRNA-sequence analysis

The miRNeasy Mini Kit (217004; Qiagen, Valencia, CA, United States) was used for miRNA extraction from red muscle samples. miRNA sequence library preparation, sequencing, mapping, and read counting by RNA-seq by Expectation-Maximization (RSEM) differential expression and analysis by DESeq2 were conducted by DNAFORM (Yokohama, Kanagawa, Japan). Fold changes and statistical differences (padj; adjusted *p*-value) in miRNA expression levels between the two groups (vehicle *versus* cisplatin, cisplatin *versus* cisplatin + 3% HET) and baseMeans are shown in the Supplementary Material. Metascape (http://metascape.org) was used for enrichment analysis of Gene Ontology (GO) biological processes (Zhou et al., 2019). miEAA (https://ccb-compute2.cs.uni-saarland.de/mieaa/) (Aparicio-Puerta et al., 2023) was used to search for target genes.

### 2.6 Immunohistology

The gastrocnemius and plantaris muscle samples were immunohistochemically stained for the slow muscle-specific myosin heavy chain 7 (MYH7) at Biopathology Institute Co., Ltd. (Oita, Japan). Briefly, muscle samples were fixed in neutral buffer solution with 10% formalin, paraffin-embedded and sectioned, and retrieved by heating for 10 min at 120°C. The sections were incubated with specific antibodies (Supplementary File) and visualized after color development using 3,3′-diaminobenzidine tetrahydrochloride solution. The cross-sectional area ratios and slow-twitch fiber counts were assessed using ImageJ. Hematoxylin and eosin staining of adjacent sections was performed at GenoStaff Co., Ltd. (Tokyo, Japan).

### 2.7 Protein expression

Muscle tissues were homogenized in RIPA buffer (Fujifilm Wako, Osaka, Japan) containing 1/100 volume of a protease inhibitor cocktail (P8340; Sigma-Aldrich, St. Louis, MO, United States) and PhosSTOP (04 906 845 001; Roche, Basel, Switzerland) and then centrifuged twice at 10,000 g at 4°C for 10 min to obtain the tissue proteins in the supernatants. Phospho- AMP-activated protein kinase (AMPK)/total-AMPK and sirtuin 1 (SIRT1) levels were assessed using Western blotting. Briefly, the samples (10 μg of total protein per lane) were subjected to SDS-PAGE, transferred onto PVDF membranes, blocked using SuperBlock (TBS) Blocking Buffer (37535; Thermo Fisher Scientific, Waltham, MA, United States) for 1 h at room temperature, and then incubated overnight at 4°C with specific primary antibodies and then secondary antibodies at room temperature for 1 h (Supplementary File). Blots were visualized using Pierce ECL Plus Western blotting Substrate (32132; GE Healthcare, Buckinghamshire, United Kingdom) and the Typhoon 9410 imager (GE Healthcare, Buckinghamshire, United Kingdom).

### 2.8 Statistical analysis

Statistical analyses were performed using GraphPad Prism version 8 (GraphPad Software, San Diego, CA, United States), R software version 4.3.1 (The R Foundation for Statistical Computing, Vienna, Austria; https://www.R-project.org/), and Stat Light 2000 (Yukms, Tokyo, Japan).

Line graphs with the mean ± standard error values were generated using GraphPad Prism, and two-way ANOVA followed by Tukey’s multiple comparisons *post hoc* test was used for statistical analysis. Box plots were generated in R using Tukey’s method. For the statistical analysis of differences between two groups, the Wilcoxon rank-sum test in R was used when outliers existed (any values that were more than 1.5 times the interquartile range above the third quartile or below the first quartile); otherwise, Student’s t-test or the Aspin–Welch t-test in Stat Light 2000 were used. For statistical analysis between multiple groups, Tukey’s multiple comparison test with a robust regression model was used in R when outliers existed (Yohai, 1987; Rabbi et al., 2022); otherwise, the Tukey–Kramer or Steel–Dwass tests in Stat Light 2000 were used. The miRNA data are displayed as MA-plots created in R. For significant differences, *p* values were calculated using the Wald method and adjusted using the Benjamini–Hochberg method. The R packages (ggbreak, ggplot2, ggrepel, MASS, multcomp, openxlsx, readxl, robust base, tidyr, and writexl) were obtained from the Comprehensive R Archive Network (CRAN). Further details are provided as Supplementary Material. Statistical significance was set at *p* < 0.05.

## 3 Results

### 3.1 Cisplatin-induced muscle atrophy in model mice

Following previous reports (Sakai et al., 2014), we established a mouse model of muscle atrophy by administering cisplatin for four consecutive days and observed the resulting induction of muscle atrophy and the subsequent recovery process. Maximum body weight decrease was observed on Day7 (*p* = 0.0003108) but recovered by Day14 to the same levels as those in the vehicle group (Figure 1B). Food intake was decreased on Day4 (*p* = 0.000666) but recovered to the same level or higher than that in the vehicle group after the cessation of cisplatin administration (Figure 1C). Hindlimb skeletal muscle (gastrocnemius and plantaris muscle) weights decreased persistently with cisplatin administration until Day14 (*p* = 0.01041) (Figure 1D), and skeletal muscle weight per gram body weight also tended to decrease on Day14 (*p* = 0.06496) (Figure 1E). Grip strength was significantly lower on Day7 (*p* = 0.006993) and Day14 (*p* = 0.04507) (Figure 1F). Thus, although the decrease in food intake was transient and the decrease in body weight had recovered on Day14, muscle atrophy and decreased muscle performance persisted.

Cisplatin-induced muscle atrophy involves both increased muscle proteolysis and decreased muscle regeneration and protein synthesis (Conte et al., 2020; Klassen et al., 2023). However, the recovery process from cisplatin-induced muscle atrophy has not been investigated in detail. Therefore, in addition to the period from Day4 and Day7, which is the acute phase of cisplatin-induced muscle atrophy, we also examined gene expression related to muscle proteolysis, synthesis, and regeneration on Day14, which can be considered as the recovery phase.

**FIGURE 2:**
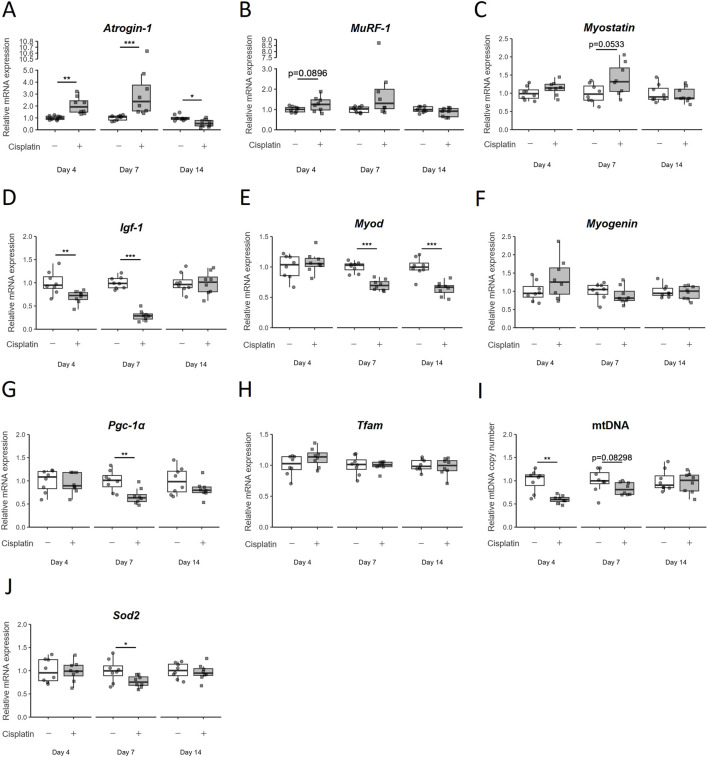
Temporal changes in the expression of various mRNA in hindlimb skeletal muscle (gastrocnemius and plantaris muscles) due to cisplatin treatment. **(A)** *Atrogin-1/muscle atrophy F-box* (*Atrogin-1*), **(B)** *Muscle RING finger 1* (*MuRF-1*), **(C)** *Myostatin*, **(D)** *Insulin-like growth factor 1* (*Igf-1*), **(E)** *Myogenic differentiation 1* (*MyoD*), **(F)** *Myogenin*, **(G)** *Peroxisome proliferator-activated receptor gamma coactivator 1α* (*Pgc-1α*), and **(H)** *Mitochondrial transcription factor A* (*Tfam*) mRNA expression, **(I)** mitochondria DNA (mtDNA) copy number, and **(J)** *Superoxide dismutase 2* (*Sod2*) mRNA expression were measured on Day4, Day7, and Day14. *n* = 8 for each group. Data are presented as Tukey’s box plots. The white box and round dots and gray box and square dots represent the vehicle and cisplatin groups, respectively. The dots represent the values for each animal. *p < 0.05, **p < 0.01, ***p < 0.001 vs vehicle (Aspin–Welch *t*-test or Wilcoxon rank-sum test).

An analysis of mRNA expression of the ubiquitin ligase family members Atrogin-1 and MuRF-1, which are directly involved in muscle atrophy, revealed that *Atrogin-1* mRNA expression was significantly upregulated by cisplatin administration on Day4 (*p* = 0.0040) and Day7 (*p* = 0.0001554), but was significantly reduced on Day14 (*p* = 0.01476) (Figure 2A). Likewise, *MuRF-1* mRNA expression also tended to be increased by cisplatin administration on Day4 (*p* = 0.0896) and Day7 (*p* = 0.1304) but recovered to the same level as that in the vehicle group on Day14 (*p* = 0.1444) (Figure 2B). We also examined the mRNA expression of *Myostatin*, a myokine that causes muscle atrophy, and found that it tended to increase on Day7 (*p* = 0.0533) but had decreased to the same level as that in the vehicle group on Day14 (*p* = 0.7000) (Figure 2C).

Next, we investigated the pathways involved in muscle regeneration and differentiation. The mRNA expression of *Insulin-like growth factor 1* (*Igf-1*), which is known to control both anabolic and catabolic pathways in skeletal muscle, was significantly decreased by cisplatin administration on Day4 (*p* = 0.00996) and Day7 (*p* = 0.0001554), but recovered to the same level as that in the vehicle group on Day14 (*p* = 1) (Figure 2D). Among the myogenic regulatory factors that regulate skeletal muscle regeneration and differentiation, *Myogenic differentiation 1* (*MyoD*) mRNA expression was continuously decreased after cisplatin administration (*p* = 0.0006216 on Day14). However, we did not observe any significant change in *Myogenin* mRNA expression (*p* = 0.7168) on Day14 (Figures 2E,F).

As cisplatin-induced myotube atrophy is reported to be related to mitochondrial damage (Matsumoto et al., 2022; Klassen et al., 2023), we assessed the expression of mitochondria-related genes in this study. Expression of *Peroxisome proliferator-activated receptor gamma coactivator 1α* (*Pgc-1α*), the master regulator of mitochondrial biogenesis, was significantly decreased on Day7 (*p* = 0.002953) in the cisplatin group but had recovered to the levels comparable to that in the vehicle group on Day14 (*p* = 0.3823) (Figure 2G). The expression of *Mitochondrial transcription factor A* (*Tfam*) mRNA was unchanged by cisplatin administration on Day7 and Day14 (*p* = 0.8785, 0.6742) (Figure 2H). The relative mtDNA copy number was lower in the cisplatin group than in the vehicle group on Day4 and Day7, but not on Day14 (*p* = 0.0013, 0.08298, and 0.7030, respectively) (Figure 2I). *Superoxide dismutase 2* (*Sod2*) mRNA localized in mitochondria decreased on Day7 (p = 0.0357), but its level on Day14 was comparable to that in the vehicle group (*p* = 0.6454) (Figure 2J). In the early stages of cisplatin-induced muscle atrophy (acute phase: Day4 and Day7), increased expression of proteolysis-related genes and decreased expression of protein synthesis-and mitochondrial biogenesis-related genes was expected based on previous reports. However, almost all changes had recovered by Day14, and only *MyoD* mRNA expression continued to decrease. This suggests that the persistence of muscle atrophy until Day14 may be related to the continued decline in *MyoD* mRNA expression.

### 3.2 Effects of HET on cisplatin-induced muscle atrophy

**FIGURE 3:**
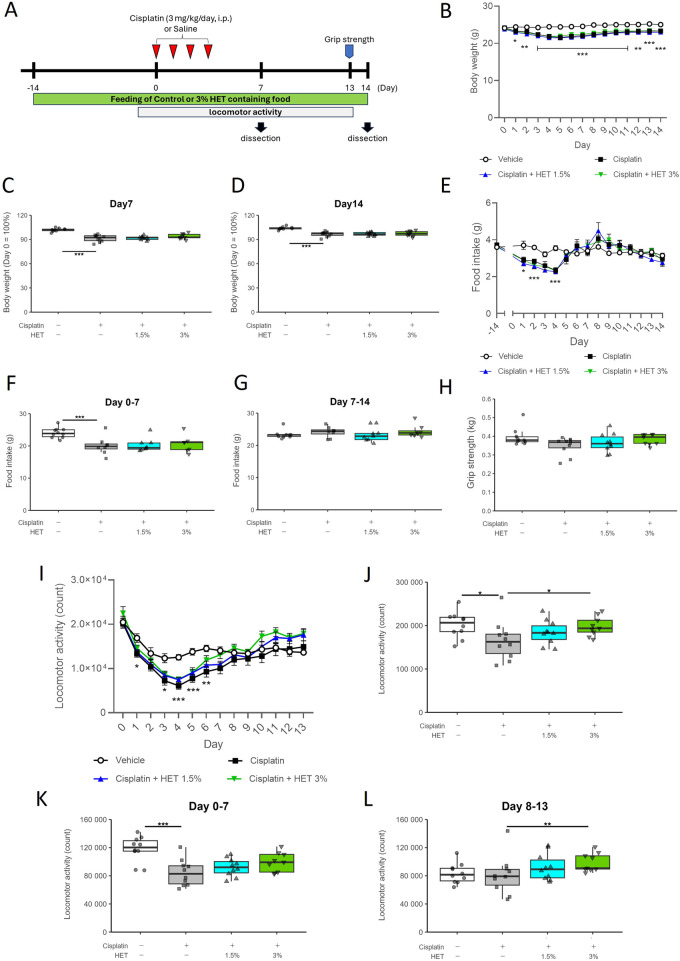
Effects of HET administration in the cisplatin-induced muscle atrophy model mice. **(A)** Experimental design. **(B)** Temporal changes in body weight, and **(C, D)** percentage of body weight change from Day0 to Day7 and Day14. *n* = 10 for the vehicle, cisplatin, and 1.5% HET groups; *n* = 9 for the 3% HET group. **(E)** Temporal change in food intake. *n* = 8–10 for each group. **(F)** Cumulative food intake in the acute phase (Day0–7). *n* = 10 for the vehicle group; *n* = 8 for the cisplatin and 1.5% HET groups; *n* = 7 for the 3% HET group. **(G)** Cumulative food intake in the recovery phase (Day7–14). *n* = 9 for the vehicle and 1.5% HET groups; *n* = 8 for the cisplatin and 3% HET groups. **(H)** Grip strength of the limb muscles was measured on Day13. The grip strength value was obtained by averaging five measurements. *n* = 10 for the vehicle, cisplatin, and 1.5% HET groups; *n* = 9 for the 3% HET group. **(I)** Temporal changes in locomotor activity, (**J–L**) cumulative locomotor activity for Day0–13 and the acute (Day0–7) and recovery (Day8–13) phases (21 h per day). *n* = 10 for the vehicle, cisplatin, and 1.5% HET groups; *n* = 9 for the 3% HET group. **(B, E, I)** Circles, squares, and upwards and downwards triangles represent the vehicle, cisplatin, 1.5% HET, and 3% HET groups, respectively. Data are presented as mean ± standard error (SE) values. *p < 0.05, **p < 0.01, ***p < 0.001 (two-way ANOVA followed by Tukey’s multiple comparisons test for *post hoc* analysis). (**C, D, F, G, H, J, K, L**) Data are presented as Tukey’s box plots. The white box and round dots, the gray box and square dots, the bule box and upwards triangles, and the green box and downwards triangles represent the vehicle, cisplatin, 1.5% HET, and 3% HET groups, respectively. The dots represent the values for each animal. *p < 0.05, **p < 0.01, ***p < 0.001 (Tukey–Kramer or Tukey’s multiple comparison test with a robust regression model).

Next, we investigated the effects of HET in the mouse model of cisplatin-induced muscle atrophy. There was no significant effect of HET on the decrease in body weight and food intake in either the acute (Day0–7) or recovery (Day7–14) phases (Figures 3B–G). We then investigated the effects of HET on grip strength and locomotor activity, which reflect skeletal muscle performance. The average grip strength in the cisplatin group was slightly lower than that in the vehicle group on Day13 (*p* = 0.755) and tended to be restored by 3% HET administration (*p* = 0.438), although the differences were not significant (Figure 3H). The cisplatin-induced decrease in locomotor activity peaked on Day4 and gradually recovered thereafter (Figure 3I). The cumulative locomotor activity for 13 days was significantly decreased in the cisplatin group compared to that in the vehicle group (*p* = 0.0293) and was significantly restored in the 3% HET group compared to that in the cisplatin group (*p* = 0.0225) (Figure 3J). The effects of HET on locomotor activity were also examined by dividing the study period into the acute (Day0–7) and recovery (Day8–13) phases. HET did not affect locomotor activity in the acute phase, and a significant increase in locomotor activity was observed only in the recovery phase (*p* = 0.00828) (Figures 3K,L). These results indicate that HET improved skeletal muscle performance without affecting body weight and food intake, suggesting that HET may not directly affect the process of induction of cisplatin-induced muscle atrophy, but rather enhances the recovery process from muscle atrophy.

**FIGURE 4:**
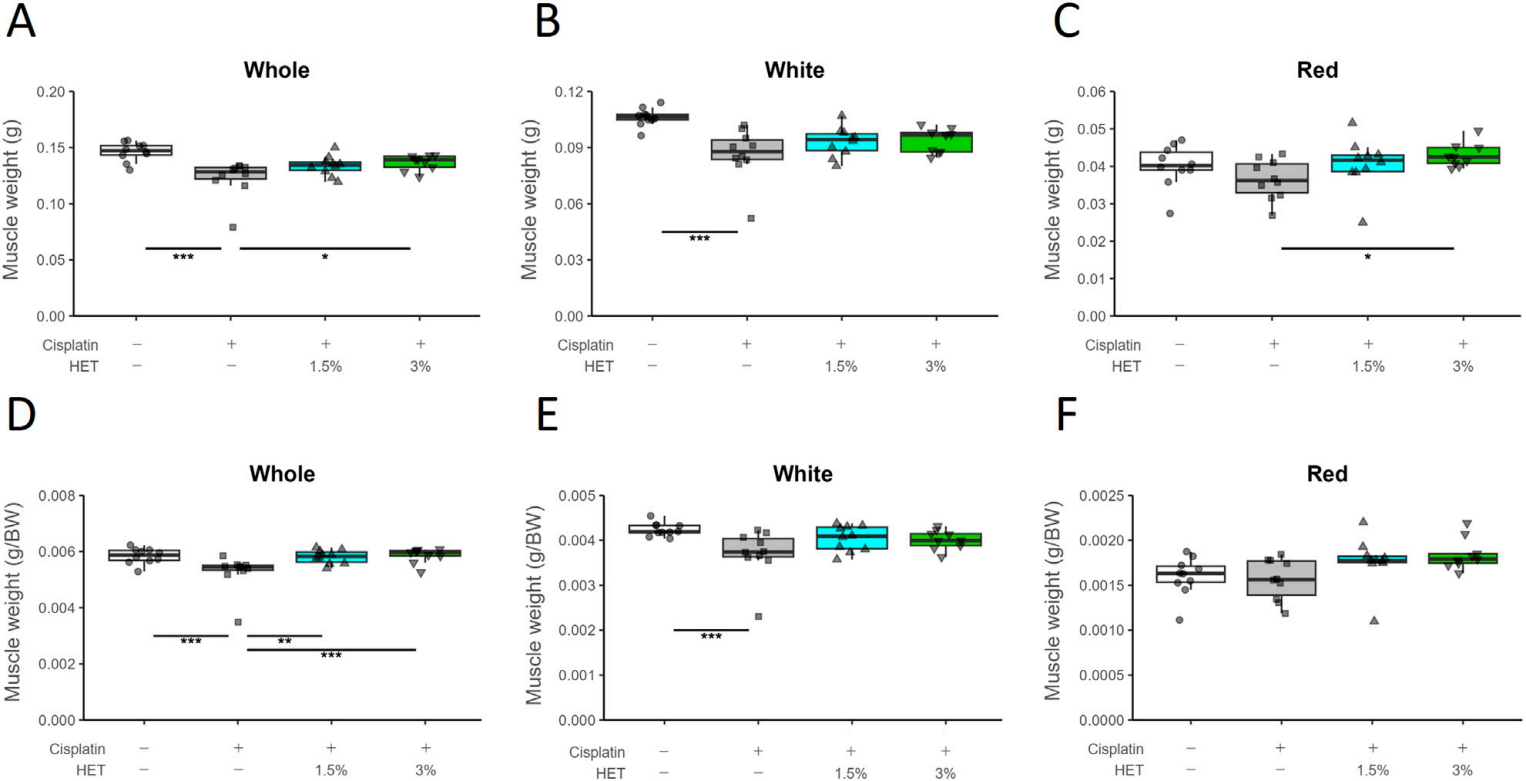
Effects of HET on muscle weight loss caused by cisplatin treatment. **(A)** Whole gastrocnemius muscle, **(B)** white region, **(C)** red region, and **(D–F)** muscle weight per gram body weight. *n* = 10 for the vehicle, cisplatin, and 1.5% HET groups; *n* = 9 for 3% HET group. Data are presented as Tukey’s box plots. The white box and round dots, the gray box and square dots, the blue box and upwards triangles, and the green box and downwards triangles represent the vehicle, cisplatin, 1.5% HET, and 3% HET groups, respectively. The dots represent the values for each animal. *p < 0.05, **p < 0.01, ***p < 0.001 (Tukey’s multiple comparison test with a robust regression model).

As skeletal muscle consists of different types of muscle fibers—fast- and slow-twitch fibers—we evaluated the effects of HET on gastrocnemius muscle weight after dividing it into two regions: the fast-twitch fiber-dominant region (white muscle) and the slow-twitch fiber-containing region (red muscle). The total weight of the gastrocnemius muscle (without the plantaris muscle) was significantly lower in the cisplatin group than in the vehicle group (*p* < 0.001) and was significantly restored in the 3% HET group (*p* = 0.0165) on Day14 (Figure 4A). The cisplatin induced-decrease in muscle weight was observed only in white muscle (*p* = <1e-04), whereas there was a trend toward a decrease in the red muscle that was not statistically significant (*p* = 0.2768); in contrast, HET increased muscle weight only in the red muscle region (*p* = 0.0129 by 3% HET) but not in the white muscle region (*p* = 0.745) (Figures 4B,C). Whole gastrocnemius muscle, red muscle, and white muscle weight per gram body weight showed the same tendency as the absolute muscle weight (Figures 4D–F). However, no increase in muscle weight caused by HET was observed on Day7 (Supplementary Figure S2). Moreover, in cisplatin-untreated mice, HET did not increase the weight of the red muscle region or locomotor activity (Supplementary Figure S3). These findings suggest that HET may restore skeletal muscle performance by acting specifically on slow-twitch fibers during the recovery from cisplatin-induced muscle atrophy, rather than during the acute phase.

**FIGURE 5:**
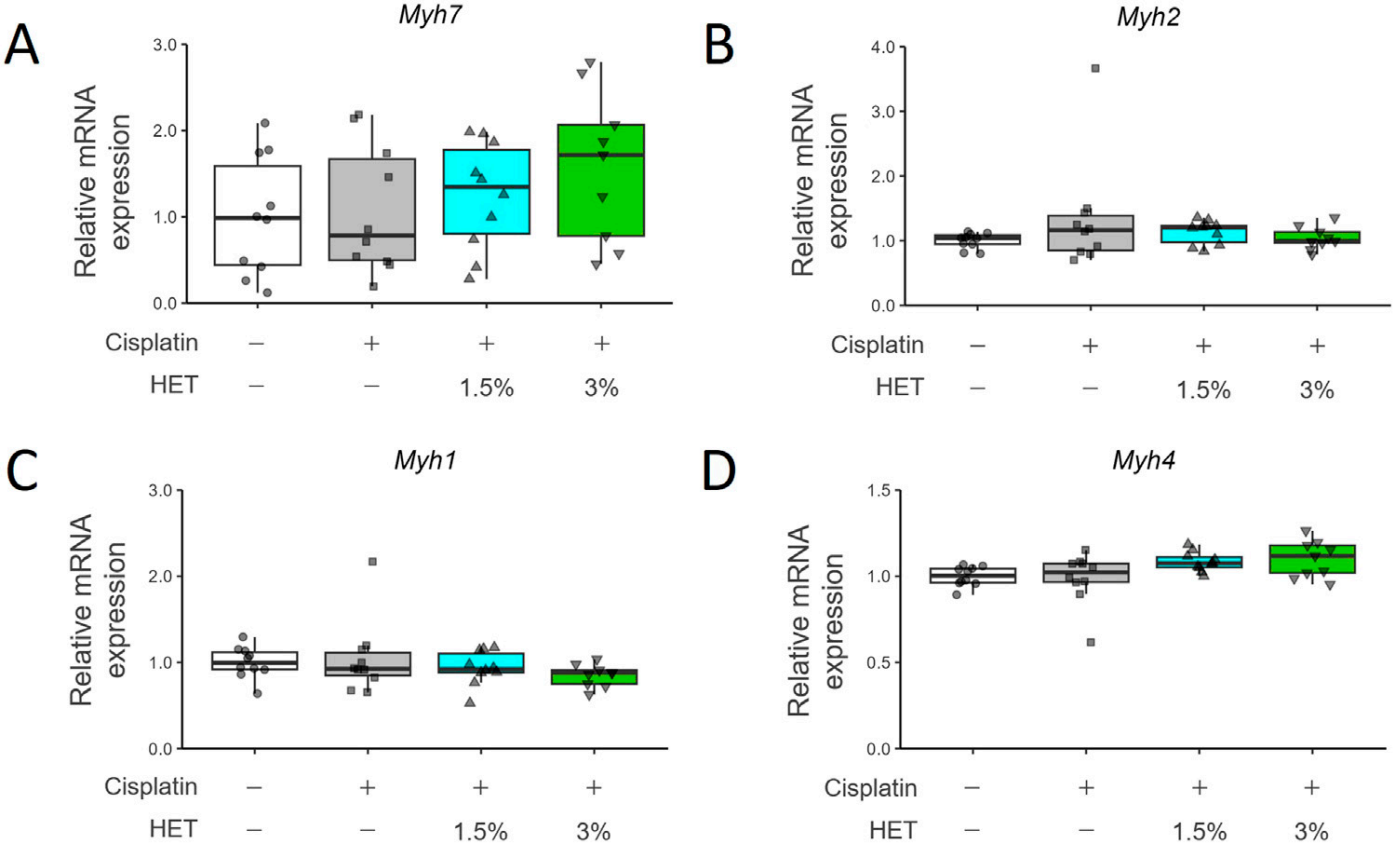
Effects of HET administration on myosin heavy chain gene expression in red gastrocnemius muscle. **(A)** *Myosin heavy chain 7* (*Myh7*), **(B)** *Myosin heavy chain 2* (*Myh2*), **(C)** *Myosin heavy chain 1* (*Myh1*), and **(D)** *Myosin heavy chain 4* (*Myh4*) mRNA expression. *n* = 10 for the vehicle, cisplatin, and 1.5% HET groups; *n* = 9 for the 3% HET group. Data are presented as Tukey’s box plots. The white box and round dots, the gray box and square dots, the blue box and upwards triangles, and the green box and downwards triangles represent the vehicle, cisplatin, 1.5% HET, and 3% HET groups, respectively. The dots represent the values for each animal. No statistically significant differences were observed between groups (Steel–Dwass or Tukey’s multiple comparison test with a robust regression model).

Since HET was suggested to affect specifically slow-twitch fibers, we then investigated its effects on mRNA expression of each myosin-heavy chain (MyHC) isoform: one slow-twitch fiber type (*Myh7/MyHCI*) and three fast-twitch fiber types (*Myh2/MyHCIIa*, *Myh1/MyHCIIx*, and *Myh4/MyHCIIb*) (Wang and Pessin, 2013) (Figures 5A–D). Cisplatin treatment did not result in any prominent changes in mRNA expression in the red muscle region; *Myh7* and *Myh4* mRNA expression tended to be slightly increased in the 3% HET group than in the cisplatin group, but the differences were not statistically significant (*p* = 0.6111 and 0.47154, respectively) (Figures 5A,D). In the white muscle region, neither cisplatin nor HET affected the expression levels of *Myh7/2/1/4* mRNAs (Supplementary Figure S4).

**FIGURE 6:**
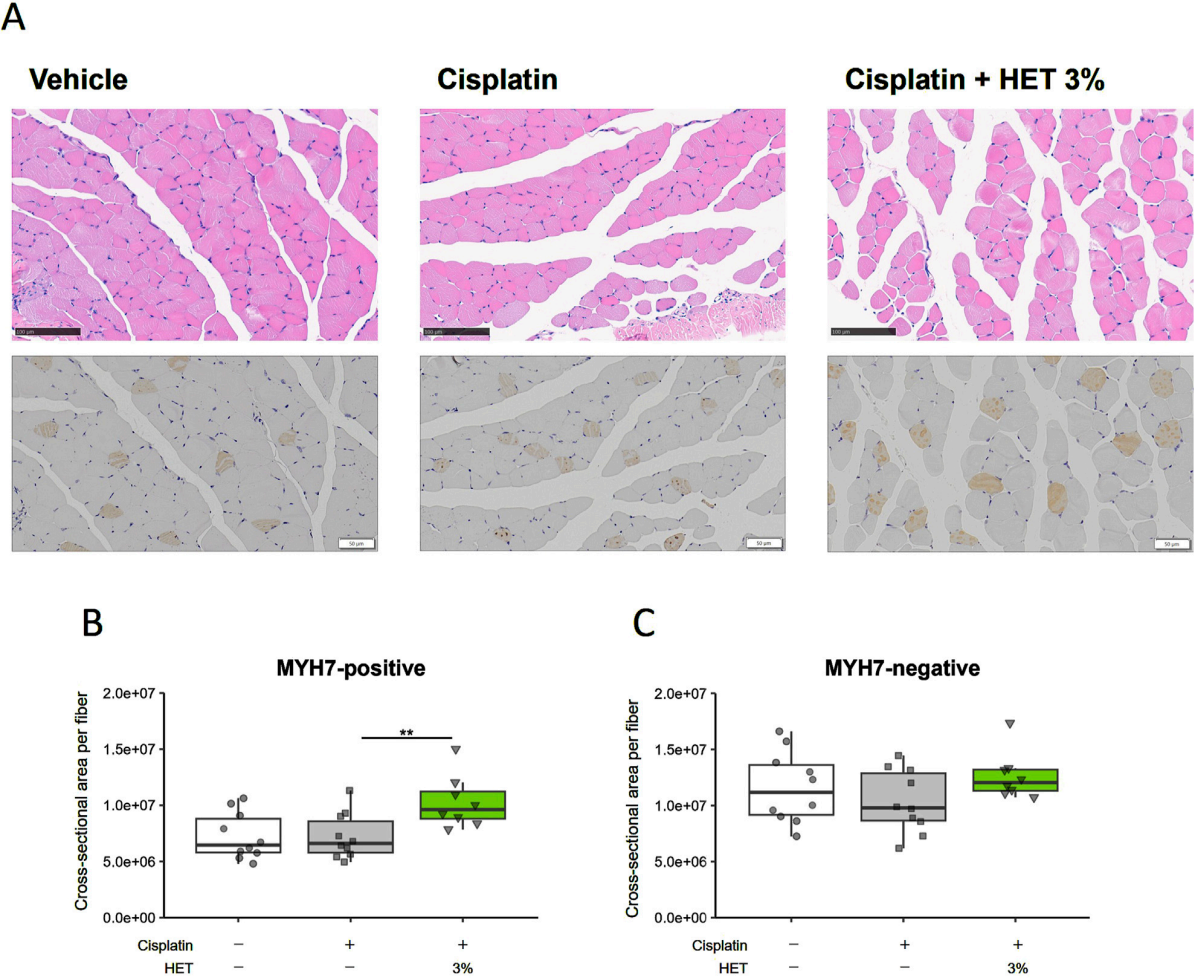
Effects of HET administration on slow-twitch fibers in the red muscle region of the gastrocnemius muscle. **(A)**, upper panel Typical hematoxylin-eosin staining images. Scale bar: 100 µm. **(A)**, lower panel Immunohistochemical staining of the slow muscle-specific myosin heavy chain protein (MYH7). Scale bar: 50 µm. Cross-sectional areas of **(B)** MYH7-positive (slow twitch fibers) and **(C)** MYH7-negative fibers. *n* = 10 for the vehicle and cisplatin groups; *n* = 8 for the 3% HET group. Data are presented as Tukey’s box plots. The white box and round dots, the gray box and square dots, and the green box and downwards triangles represent the vehicle, cisplatin, and 3% HET groups, respectively. The dots represent the values for each animal. **p < 0.01 (Tukey’s multiple comparison test with a robust regression model).

We also assessed the protein levels of MyHCI (MYH7) using immunohistochemical staining (Figure 6A, lower panel). Typical images of hematoxylin and eosin staining are shown in the upper panel of Figure 6A. The MYH7-positive cross-sectional area was unchanged by cisplatin administration but significantly increased by 3% HET administration (*p* = 0.0075) (Figure 6B). In contrast, the MYH7-negative cross-sectional area was not changed by cisplatin or 3% HET administration (Figure 6C). Thus, although the increase in total expression of *Myh7* mRNA in the red muscle region was not significant, HET appeared to increase the cross-sectional area of slow-twitch muscle fibers. These results suggest that HET enhances the recovery process from cisplatin-induced muscle damage mainly by increasing the mass of slow-twitch fibers in the mouse hindlimb.

**FIGURE 7:**
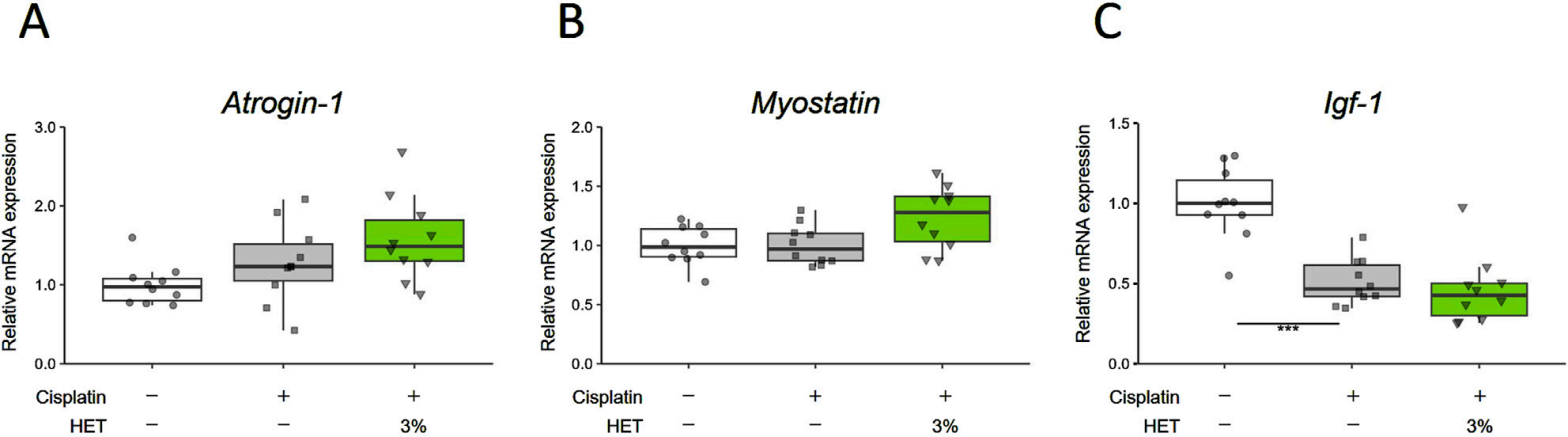
Effects of HET on muscle proteolysis and synthesis on Day7. **(A)** *Atrogin-1/muscle atrophy F-box* (*Atrogin-1*), **(B)** *Myostatin*, and **(C)** *Insulin-like growth factor 1* (*Igf-1*) mRNA expression in red muscle regions. *n* = 10 for each group. Data are presented as Tukey’s box plots. The white box and round dots, the gray box and square dots, and the green box and downwards triangles represent the vehicle, cisplatin, and 3% HET groups, respectively. The dots represent the values for each animal. ***p < 0.001 (Steel–Dwass or Tukey’s multiple comparison test with a robust regression model).

Next, we investigated why HET specifically affected slow-twitch fibers in cisplatin-induced muscle atrophy. First, we examined the effects of HET on *Atrogin-1*, *Myostatin,* and *Igf-1*, which were altered by cisplatin in the acute phase (Day7), but no obvious effects of HET were observed on any of these in the red muscle regions (*p* = 0.6144, 0.1025, and 0.414) (Figures 7A–C). We also investigated the effect of HET on *MyoD* mRNA levels, since a sustained decrease in *MyoD* mRNA expression was assumed to be involved in the continued atrophy of the white muscle region in this model (Figures 2E, 4B,E). *MyoD* mRNA expression was also significantly decreased by cisplatin administration in white muscle (*p* = 0.0343), but there was no change in the red muscle region (*p* = 0.236) on Day14 (Supplementary Figure S5). Furthermore, HET did not affect the expression levels of *MyoD* in either region (Supplementary Figure S5). The absence of any observed change in expression in the red muscle regions after HET administration (*p* = 0.995) indicates that *MyoD* expression is not involved in the effect of HET increasing red muscle weight.

**FIGURE 8:**
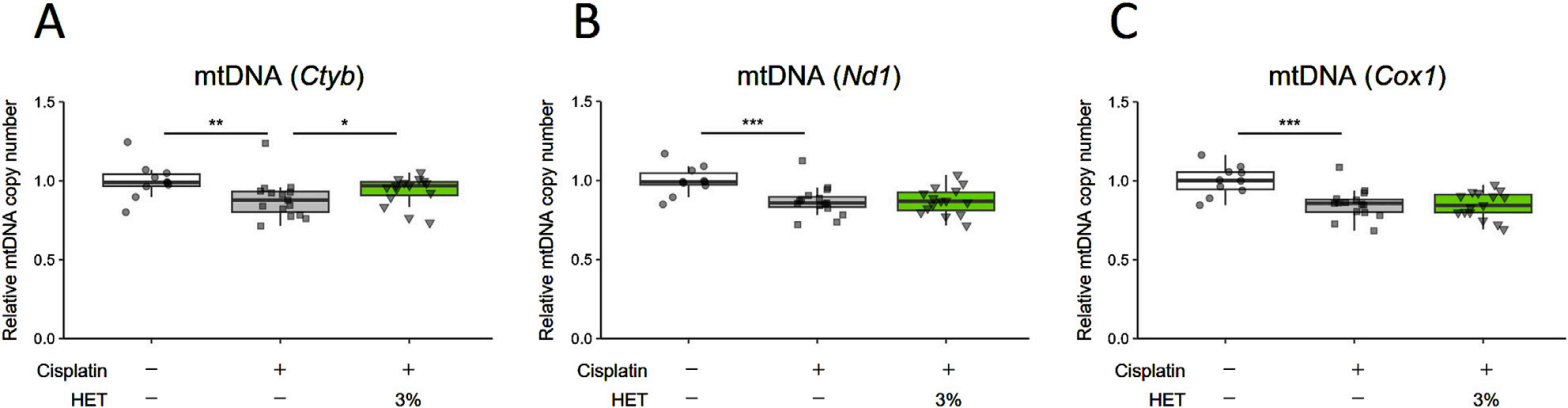
Effects of HET administration on mitochondrial DNA copy number in red muscle regions. **(A)** Cytochrome b (*Cytb*), **(B)** NADH dehydrogenase subunit 1 (*Nd1*), and **(C)** cytochrome c oxidase subunit 1 (*Cox1*) mtDNA copy numbers. *n* = 10 for the vehicle group; *n* = 15 for the cisplatin and 3% HET groups. Data are presented as Tukey’s box plots. The white box and round dots, the gray box and square dots, and the green box and downwards triangles represent the vehicle, cisplatin, and 3% HET groups, respectively. The dots represent the values for each animal. *p < 0.05, **p < 0.01, ***p < 0.001 (Tukey’s multiple comparison test with a robust regression model).

Myogenin, myocyte enhancer factor-2c (Mef2c), Nuclear factor of activated T cells 1 (Nfatc1), and Class Ⅱ histone deacetylase 4 (HDAC4) are known to be involved in the regulation of slow-twitch fiber differentiation and muscle hypertrophy (Hughes et al., 1993; Potthoff et al., 2007; Alexander et al., 2010; Ehlers et al., 2014; Anderson et al., 2015). Therefore, we examined the effects of HET on the expression of their mRNAs. Contrary to our expectations, we did not observe any corresponding significant changes in the red muscle regions after administration of 3% HET (*p* = 0.9998, 0.995, 1.000 and 0.878) (Supplementary Figure S6). Moreover, activation of the AMPK/SIRT1/PGC-1α signaling pathway has been reported as a mechanism by which polyphenols and arginine induce fiber type transformation from type II to type I (Chen et al., 2018; Wen et al., 2020; Xue et al., 2021); thus, we also examined the effects of HET on phospho-AMPK and SIRT1 protein levels, but did not observe any clear effect on them on Day14 (Supplementary Figure S7). As mitochondrial dysfunction has been reported to be involved in cisplatin-induced muscle atrophy (Sirago et al., 2017; Seo et al., 2021; Matsumoto et al., 2022), we then examined the effects of HET on the expression of mitochondria-related genes. The expressions of *Pgc-1α, Tfam*, *Sirt1*, *Sirt3*, *Nuclear respiratory factor 1* (*Nrf1*), and *Sod2* mRNA were also not significantly different between groups and remained unchanged by HET administration (p = 0.962, 0.900, 0.812, 0.9948, 0.975, and 0.9614 between the cisplatin and cisplatin + 3% HET groups) (Supplementary Figure S8). Administration of 3% HET significantly increased (*p* = 0.01609) the decrease in mtDNA level of cytochrome b (*Cytb*) (*p* = 0.00226) in the red muscle, but not of the copy numbers for NADH dehydrogenase subunit 1 (*Nd1*) and cytochrome c oxidase subunit 1 (*Cox1*) (*p* = 0.947093 and 0.999047) (Figures 8A–C).

### 3.3 miRNA-sequence analysis

As mentioned earlier, we did not find any definite HET-evoked changes in the expression levels of key regulators of skeletal muscle fiber-type, i.e., MyoD, Myogenin, Myostatin, MEF2, NFAT, HDAC4, SIRT1, and PGC-1α and attempts to identify possible mechanisms by which HET can specifically affect slow-twitch fibers based on the findings of previous reports did not yield any definitive answers. Therefore, we focused on miRNAs known to play a role in fine-tuning gene expression and translation in eukaryotes. In addition to myogenic regulatory factors, growth factors, hormones, and myokines, some miRNAs are not only reportedly involved in skeletal muscle development and muscle-related diseases, but also in defining muscle fiber types. Thus, we performed miRNA-seq analysis using the red muscle region of the gastrocnemius muscle, where the effect of HET was most prominent.

**FIGURE 9:**
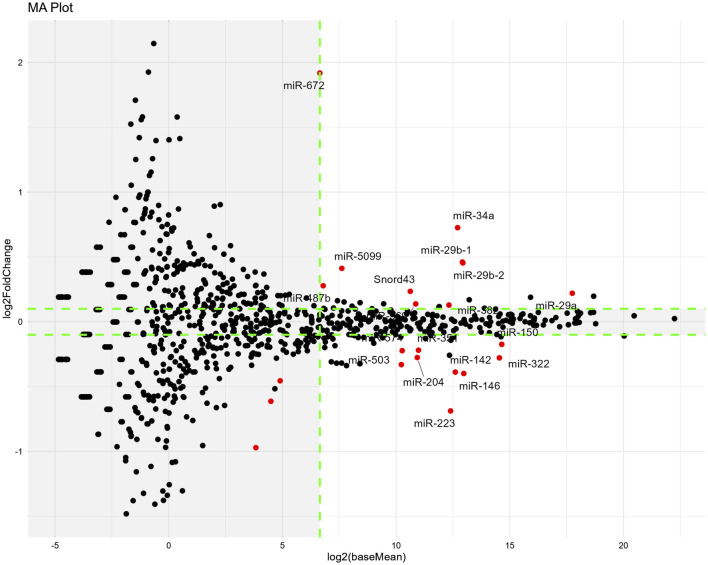
Changes in miRNA expression induced by cisplatin administration on Day14. The miRNA-seq analysis was performed using red muscle region sample. MA-plots show the differences in miRNA expression between the vehicle and cisplatin groups. *n* = 10 for the vehicle group; *n* = 15 for the cisplatin groups. MA-plots were generated by plotting Log2FoldChange values on the y-axis and Log2(baseMean) values on the x-axis (values obtained from the DESeq2 data; Supplementary Material). MiRNAs that were significantly different (padj <0.05) between two groups are represented by red dots. Tagged miRNAs meet the following thresholds: padj <0.05, absolute value of Log2FoldChange >0.1, and baseMean >100. Outside the threshold, the background is shaded in gray. Log2FoldChange, difference in expression levels between the comparison groups; baseMean, mean of normalized counts of all samples from the two groups being compared. Padj; adjusted *p*-value.

From the obtained DESeq2 data (Supplementary File), we extracted miRNAs of note according to the following threshold levels: padj <0.05, absolute Log2FoldChange >0.1, and baseMean >100. Based on a comparison of the vehicle *versus* cisplatin groups (Figure 9), we extracted nine upregulated miRNAs and nine downregulated miRNAs by cisplatin administration (a total of 18 miRNAs from 1003 miRNAs listed, Table 1). Gene Ontology (GO) analysis was performed using Metascape (https://metascape.org/) to identify the predicted functions of the miRNAs and the potential effects of cisplatin. As a result (Supplementary Figure S8), the miRNAs upregulated by cisplatin showed significant associations with long-term synaptic potentiation (GO:0060291), cellular response to leukemia inhibitory factor (GO:1990830), sensory perception of sound (GO:0007605), and cellular response to phenylalanine (GO:0071234). In contrast, the miRNAs downregulated by cisplatin were significantly associated with cellular response to leukemia inhibitory factor (GO:1990830), response to oxygen levels (GO:0070482), cellular response to lipid (GO:0071396), and cellular response to organic cyclic compound (GO:0071407).

**TABLE 1:** Extracted miRNA (vehicle vs. cisplatin).

Cisplatin	GeneName	baseMean	log2FoldChange	padj
UP	miR-29a	219821	0.2202	1.2345E-03
	miR-29b-2	7837	0.4539	6.3194E-08
	miR-29b-1	7715	0.4604	5.9283E-08
	miR-34a	6675	0.7260	5.1073E-15
	miR-382	5124	0.1289	1.3373E-02
	miR-369	1858	0.1380	1.6045E-03
	miR-5099	196	0.4120	8.2151E-04
	miR-487b	111	0.2779	4.8836E-02
	miR-672	100	1.9186	1.2265E-45
DOWN	miR-150	25626	−0.1740	2.5523E-02
	miR-322	23846	−0.2780	1.9055E-05
	miR-146	8050	−0.3984	5.7175E-07
	miR-142	6220	−0.3880	9.0689E-05
	miR-223	5380	−0.6875	3.6453E-21
	miR-351	2024	−0.2192	7.4954E-04
	miR-204	1950	−0.2757	1.2727E-07
	miR-574	1231	−0.2229	7.1304E-03
	miR-503	1200	−0.3303	2.6790E-10

We then compared our results to those of a previous study (Jang et al., 2021). We used miEAA to predict the target genes of the extracted miRNAs in our study and investigated whether the expression of those target genes was suppressed by cisplatin administration based on RNA-seq data in that study (Jang et al., 2021), in which row gene expression data (GSE147613) for Cisplatin and Normal were obtained from the Gene Expression Omnibus dataset of the National Center for Biotechnology Information (https://www.ncbi.nlm.nih.gov/geo) (Jang et al., 2021).

Of the 28 target genes predicted from the list of miRNAs with increased levels in our study (Supplementary File), most were also downregulated after cisplatin administration in a previous report (Jang et al., 2021). In contrast, miEAA predicted only *Il-6* mRNA as the target gene of the miRNA that had reduced levels after cisplatin administration in our study, but its level was increased in the previous study and remained unchanged in our study (Supplementary Figure S10). This was probably because some of miRNAs with increased levels in our study were also predicted to target IL-6. Among the miRNAs increased by cisplatin, those that had large baseMean values were miR-29a/b and miR-34a*.* Thus, changes in their expression levels were estimated to have large effects.

**FIGURE 10:**
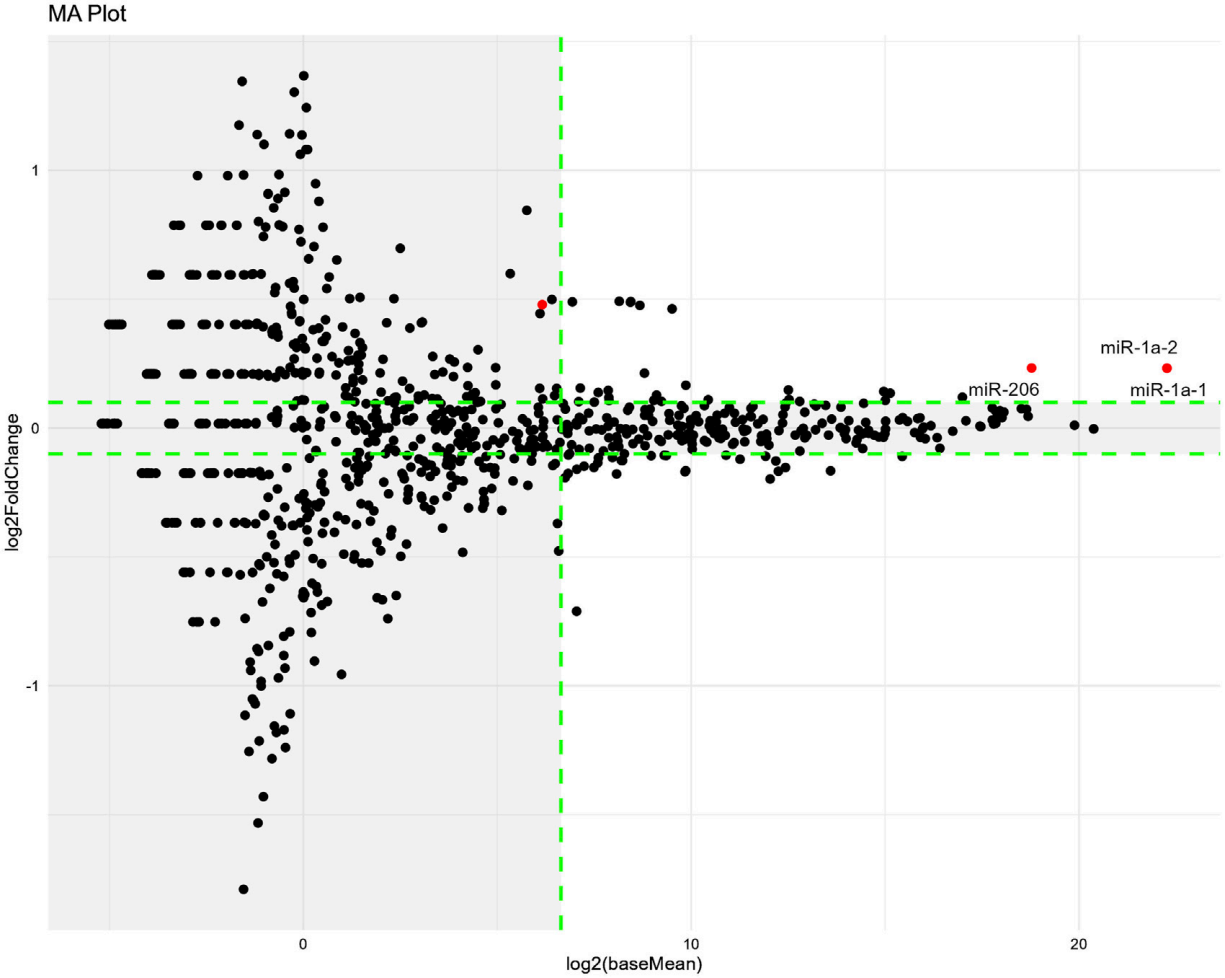
Changes in miRNA expression induced by HET administration on Day14. The miRNA-seq analysis was performed using red muscle region samples. MA-plots show the differences in miRNA expression between the cisplatin and 3% HET groups. *n* = 15 for the cisplatin and 3% HET groups. MA-plots were generated by plotting Log2FoldChange values on the y-axis and Log2(baseMean) values on the x-axis (values obtained from the DESeq2 data; Supplementary Material). MiRNAs that were significantly different (padj <0.05) between two groups are represented by red dots. Tagged miRNAs meet the following thresholds: padj <0.05, absolute value of Log2FoldChange >0.1, and baseMean >100. Outside the threshold, the background is shaded in gray. Log2FoldChange, difference in expression levels between the comparison groups; baseMean, mean of normalized counts of all samples from two groups being compared. Padj; adjusted *p*-value.

Regarding the difference in miRNA expression between the cisplatin and cisplatin + 3% HET groups, three miRNAs (miR-1a-1, miR-1a-2, and miR-206*,* which facilitate satellite cell proliferation and differentiation) were extracted according to our threshold levels of the 1023 miRNAs (Figure 10; Table 2) (Chen et al., 2010; Salant et al., 2020). They were significantly upregulated by HET administration and showed the highest baseMean values, indicating large effects. As miR-206 has been reported to be involved in determining the slow muscle fiber type (Bjorkman et al., 2020), it may have played a role in the thickening of slow fiber muscle in the cisplatin + HET groups. By searching the miEAA database, we identified 70 experimentally validated target genes for miR-206 or miR-1 (Supplementary File). Among them, only HDAC4 has been reported to influence the induction of slow-twitch muscle fibers by suppressing the transcriptional activity of MEF2 (Potthoff et al., 2007). Therefore, HDAC4 was considered a plausible target gene of miR-206 or miR-1, which was upregulated by HET administration. However, in this study, no change in *Hdac4* mRNA levels was observed following HET administration (Supplementary Figure S6D).

**TABLE 2:** Extracted miRNA (cisplatin vs. cisplatin + 3% HET).

Cisplatin +3% HET	GeneName	baseMean	log2FoldChange	padj
UP	miR-1a-1	5054612	0.2324	1.8356E-02
	miR-1a-2	5053076	0.2324	1.8356E-02
	miR-206	449833	0.2332	2.1912E-02

## 4 Discussion

In this study, we demonstrated that 1) recovery from cisplatin-induced muscle atrophy is specifically delayed in the “white muscle” regions of the gastrocnemius muscle, probably due to the persistent decrease in *MyoD* mRNA expression and that HET treatment 2) improved reduced locomotor activity, increased gastrocnemius muscle weight, especially in the “red muscle” region, and increased the thickness of slow-twitch muscle fibers and 3) upregulated three miRNA involved in skeletal muscle development, differentiation, and regeneration.

### 4.1 Cisplatin-induced muscle atrophy continued for prolonged periods

Multiple lines of evidence indicate that cisplatin causes skeletal muscle atrophy by both activating muscle protein degradation and downregulating protein synthesis and muscle regeneration systems (Conte et al., 2020; Klassen et al., 2023). However, the recovery process is yet to be studied precisely. Since recovery of body and muscle weight after cisplatin administration takes approximately 4–6 weeks (Garcia et al., 2013; Hojman et al., 2014), we conducted a detailed pathophysiological evaluation of the recovery phase in addition to the acute phase after cisplatin administration.

Our results indicate that cisplatin regulates specific genes to induce muscle atrophy by enhancing the protein degradation system, decrease protein synthesis, disrupt mitochondrial biogenesis and reduced mtDNA level in the acute phase (Day4 to Day7), and these findings are in good agreement with previous reports (Sirago et al., 2017; Seo et al., 2021; Matsumoto et al., 2022). Although these changes were found to be almost completely restored on Day14, the decrease in skeletal muscle weight and grip strength persisted. One possible explanation for this apparent discrepancy may be the persistent suppression of *MyoD* expression. MyoD is essential not only for myogenesis, but also for skeletal muscle regeneration, and several reports have shown that *MyoD* is downregulated during the acute phase after cisplatin administration (Chen et al., 2015; Wu et al., 2019; Jang et al., 2021). Furthermore, previous studies have indicated that MyoD is expressed at higher levels in fast-twitch fibers than in slow-twitch fibers (Hughes et al., 1997) and that its overexpression in muscle results in a slow-to-fast fiber type conversion (Ekmark et al., 2007), which is also in line with our results. Notably, our study showed, for the first time, that the decrease in *MyoD* expression persisted until Day14 after cisplatin administration, and this may be one of the reasons why the recovery of skeletal muscle weight and grip strength was delayed in cisplatin-induced muscle atrophy.

### 4.2 HET ameliorates muscle performance in cisplatin induced muscle atrophy model

Our analysis showed that body weight and food intake were unaffected by HET administration in both the acute and recovery phases in our model of cisplatin-induced muscle atrophy. When we investigated the effects of HET on factors that reflect muscle performance, we found that the decreased average grip strength was partially recovered by HET administration, although the difference was not statistically significant. Moreover, the cisplatin-induced decrease in locomotor activity peaked on Day4 and gradually recovered thereafter. Although HET did not affect locomotor activities in the acute phase (Day0–7), it specifically increased them in the recovery phase (Day8–13), indicating that HET does not affect the induction of muscle atrophy, but rather enhances the recovery process. Since the induction of skeletal muscle hypertrophy has been reported to increase muscle performance (Dyle et al., 2014; Yoo et al., 2021), we assumed that restoration of gastrocnemius muscle weight by HET administration led to the restoration of decreased locomotor activity in this study. On the other hand, voluntary exercise prevented cisplatin-induced muscle wasting in mice and locomotive wheel running activity increased the weight of the gastrocnemius muscle in disuse muscle atrophy model mice (Hojman et al., 2014; Brooks et al., 2018) and endurance exercise strengthened slow-twitch muscles (Crupi et al., 2018). Thus, there is a possibility that HET might have increased locomotor activity and then restored skeletal muscle atrophy since it reportedly improves physical activity in elderly patients (Watanabe and Maeda, 2022) and aged mice (Nahata et al., 2021; Nahata et al., 2022). However, further investigations are required to clarify the precise relationships involved. Moreover, since the anxiolytic effect of cisplatin can reduce motor activity-related parameters (Vukovic et al., 2019) and HET improved avoidance behavior in a depression model (Tohda and Mingmalairak, 2013) and anxiety-like behavior in lipopolysaccharide-treated mice (Ushio et al., 2022), it is possible that the improvement of anxiety by HET led to an additional increase in locomotor activity.

### 4.3 The effects of HET are specific to slow-twitch fibers

The gastrocnemius muscle consists of both slow- and fast-twitch fibers and can be divided into two different regions: the “red muscle” region, characterized by the presence of “slow” isoforms of contractile proteins and a high oxidative enzyme content, and the “white muscle” region, which has a predominance of glycolytic enzymes and “fast” isoforms of contractile proteins (Burkholder et al., 1994; Glancy and Balaban, 2011; Dimauro et al., 2019; Lloyd et al., 2023). Thus, we evaluated the effect of HET by dividing the gastrocnemius muscle into red and white regions.

We found that, although the decrease in the weight of white muscle was unaffected, the weight of red muscle (containing slow-twitch fibers) was significantly increased by HET administration, indicating that HET specifically affected slow-twitch fibers in the red muscle region. To confirm this, we investigated the effects of HET on subtype-specific gene and protein expression patterns. Although there was no definite change in the mRNA expression levels of myosin heavy chain subtypes, including slow fiber-specific *Myh7*, immunohistochemical analyses showed that MYH7-positive slow-twitch fibers were significantly increased following HET treatment.

Grip strength is primarily considered to reflect the function of fast-twitch muscles (Song et al., 2021), whereas locomotor activity is more indicative of endurance, which reflects the function of slow-twitch muscles (Crupi et al., 2018). The insufficient recovery of grip strength even 2 weeks after cisplatin administration in this study is believed to be due to the continued atrophy of fast-twitch muscles, probably caused by the sustained decrease in *MyoD* levels. Cisplatin has been reported to cause disruption of neuromuscular junctions (Huot et al., 2022) which has been implicated in the pathogenesis of fast-twitch muscle-dominant atrophy observed in several neuromuscular disorders such as Duchenne muscular dystrophy, amyotrophic lateral sclerosis and sarcopenia (Lloyd et al., 2023). Thus, white muscle region may be more susceptible to cisplatin and resulted in the weight decrease. In contrast, locomotor activity naturally recovered after Day8, and HET further increased this activity which was consistent with the increase in slow-twitch muscle area. Thus, the changes in skeletal muscle performance reflected the changes in muscle fiber types.

### 4.4 Comparison with the known mechanism of HET

We then investigated the mechanism by which HET specifically exerts its action on slow-twitch fibers but found no effect of HET on genes that were altered by cisplatin in the acute phase, such as muscle atrophy- and mitochondria-related genes. Additionally, HET did not restore the decreased *MyoD* mRNA expression, which is likely involved in persistent white muscle atrophy. Furthermore, HET administration did not lead to any significant changes in the gene expressions, that are involved in the differentiation and hypertrophy of slow-twitch fibers.

With regard to other models of skeletal muscle atrophy, HET has been reported to improve muscle atrophy by suppressing Atrogin-1 and activating AMPK in a disuse-induced muscle atrophy model (Yakabe et al., 2022), by suppressing IL-6 production in colon 26 adenocarcinoma (Yae et al., 2012), and inhibiting inflammation and oxidative stress in an amyotrophic lateral sclerosis mouse model (Cai and Yang, 2019). However, we did not detect any changes in the expression of these factors following HET administration in this study. Thus, different mechanisms were assumed to be involved in the effects of HET in this study.

All previous studies on the effects of drugs on cisplatin-induced muscle atrophy have focused on their preventive and therapeutic effects in the acute phase of muscle atrophy. In contrast, the newly discovered effects of HET were limited to the recovery phase in this study. Furthermore, HET had no effect on normal mice and its effect was observed only after cisplatin induced muscle injury. This suggests that HET specifically acts during the recovery process rather than nonspecifically affecting the muscles. Since activation, proliferation, and differentiation of skeletal muscle satellite cells, which are important for muscle regeneration, are reportedly affected by fibro/adipogenic progenitors, inflammatory/immune cells, etc., (Koike et al., 2022; Jiang et al., 2024), their involvement in the action mechanism of HET is also highly intriguing and requires future investigation.

### 4.5 Changes in skeletal muscle miRNA expression

We also attempted to determine the roles of the major regulators of skeletal muscle fiber-type with respect to the slow-twitch fiber-specific effects of HET. However, contrary to our expectations, we did not observe any changes in this regard, and focused on miRNAs. Recently, several miRNAs, including muscle-specific miRNAs (myomiRs), have been shown to significantly affect skeletal muscle differentiation, regeneration, atrophy, and functioning (Horak et al., 2016). Furthermore, some myomiRs (i.e., miR-1a-1/2, miR-133a/b, miR-206, miR-208b, miR-486, and miR-499) are known to be involved in skeletal muscle fiber-type regulation (van Rooij et al., 2009; Zhang et al., 2014; Bjorkman et al., 2020). Thus, we performed a comprehensive miRNA-sequence analysis to identify the miRNAs that may be related to the mechanism of action of HET.

Cisplatin treatment increased the levels of nine miRNAs and the predicted target genes were in good agreement with the changes in mRNA levels in previous study by Jang et al. (2021). This suggests that some of these miRNAs altered the expression levels of several mRNAs in skeletal muscle and may have thus contributed to the induction and/or regression of skeletal muscle atrophy.

Among the nine upregulated miRNAs that were significantly upregulated by cisplatin, miR-29a, miR-29b, and miR-34a appear to be particularly important, because their expression levels were much higher than those of the other miRNAs. miR-29a and miR-29b belong to the miR-29 family and have been reported to either suppress skeletal muscle atrophy by inhibiting MuRF-1, Ying Yang-1, and PTEN (Wang et al., 2011; Wang et al., 2020; Liu et al., 2021) or promote it by inhibiting IGF-1 and PI3K (p85α) (Li et al., 2017), respectively. Considering that the observed expression levels of miR-29a were much higher than that of miR-29b in our study, it is possible that the miR-29 family facilitated the overall recovery from muscle atrophy. Furthermore, both miR-29 and miR-34a significantly increase with age in the skeletal muscle and are senescence-associated miRNAs (Hu et al., 2014; Fulzele et al., 2019; Alfonzo et al., 2022), implying that their increase might have induced senescence-like conditions in the skeletal muscle after cisplatin administration.

HET administration significantly upregulated the expression of three miRNAs: miR-1a-1, miR-1a-2, and miR-206*.* Both miR-1 and miR-206 are increased after muscle damage and enhance the differentiation of skeletal muscle satellite cells by targeting Pax7 (Chen et al., 2010) and the induction of slow-twitch muscle by targeting HDAC4 (Potthoff et al., 2007), and were highly expressed in the red muscle region and increased by HET administration in this study; thus, we assumed that both effects were involved in increasing the weight of the red muscle region. MiR-1 is the most abundant miRNA in the heart tissue, accounting for 40% of the total miRNAs (Rao et al., 2009) and has been shown to play a critical role in heart development (Wei et al., 2014; Luo et al., 2018). In contrast, miR-206 is involved in skeletal muscle satellite cell proliferation and differentiation (Chen et al., 2010), regeneration of neuromuscular synapses by targeting HDAC4 (Williams et al., 2009) and repairment of neuromuscular junctions after nerve injury (Valdez et al., 2014). Moreover, miR-206 is highly expressed in slow-twitch soleus muscles (Siracusa et al., 2018), and its deficiency delays differentiation and formation of myotube (Salant et al., 2020). Moreover, as the cross-sectional area of type I (slow-twitch) myofibers in the soleus muscle of male miR-206-knockout mice (Bjorkman et al., 2020) was lower, miR-206 may have played a role in thickening the slow fiber muscles in the HET group in this study, indicating its involvement in the slow-twitch fiber-specific action mechanism of HET. Because miR-206 is important for mitochondrial and muscle function (Przanowska et al., 2020), it may also be involved in the increase in locomotor activity.

Based on the above findings, it is plausible that HET administration increases slow-twitch muscle fibers through the upregulation of miR-1 and/or miR-206. To further elucidate the underlying mechanism, we searched for target genes of miR-1 and/or miR-206 using the miEAA database. Although we identified 70 experimentally validated target genes for miR-1/miR-206, only HDAC4 has been reported to influence the regulation of slow-twitch muscle fibers by suppressing the transcriptional activity of MEF2 (Potthoff et al., 2007). Therefore, HDAC4 was considered a plausible target gene of miR-1/miR-206, which is upregulated by HET administration. However, in this study, no changes in *Hdac4* mRNA levels were observed following HET administration. This suggests that the inhibition of *Hdac4* translation rather than transcription may be involved, that target genes other than *Hdac4* may play a role in the regulation of slow muscle fibers by HET, or that subtle changes in multiple target genes may collectively contribute to the induction of slow-twitch muscle differentiation/ regeneration.

### 4.6 Limitations

This study has several limitations. First, although we found that HET treatment increased slow-twitch muscle fibers in the red region of gastrocnemius muscle and significantly increased miR-1/miR-206 in the same area, we could not establish a direct causal relationship between the two. To directly demonstrate this experimentally, it would be necessary to knock down miR-1/miR-206 using miRNA sponges or anti-microRNAs (Winkle et al., 2021) or to use muscle-specific miR-206-deficient mice (Williams et al., 2009; Bjorkman et al., 2020). Second, this study was unable to identify the target genes of miR-1 and miR-206 involved in slow muscle induction. To address this, a comprehensive analysis of both miRNAs and mRNAs will be necessary in the future to elucidate their mutual relationship. Third, although we demonstrated the positive effects of HET overall, we could not specifically determine which of the 10 crude drugs or their metabolites were involved in the restorative effects of HET on cisplatin induced-muscle atrophy in our model. Several reports have indicated the possibility of some of the metabolites of HET improving muscle atrophy and locomotor activities, specifically astragaloside Ⅳ from Astragalus root (Jiang et al., 2020), compound K (Kim et al., 2022) and Ginsenoside Rd (Wijaya et al., 2022) from ginseng, Saikosaponin A and D from Bupleurum root (Huang M. et al., 2023), ursolic acid from jujube (Tao et al., 2023), and nobiletin (Nohara et al., 2019; Wang et al., 2023) and naringin (Lv et al., 2023) from Citrus unshiu peel. In the future, it will be important to clarify which of these metabolites contribute to the effect of HET on muscle atrophy through *in vitro* studies using muscle cells, such as C2C12 and other approaches. Lastly, miRNA expression was investigated using only the red muscle region, although its weight was not decreased by cisplatin on Day14. Thus, further investigation of the changes in miRNA expression in white muscle will be needed in future studies to better understand the effects of cisplatin-induced muscle atrophy.

## 5 Conclusion

Cisplatin-induced muscle atrophy involved induction of proteolysis, reduced protein synthesis, and mitochondrial damage in the acute phase and a continuous decrease in *MyoD* levels during the recovery phase. HET increased locomotive activity and muscle weight, probably by enhancing myogenesis/regeneration of slow-twitch fibers, which was related to increased miR-1 and miR-206 expression. These findings indicate that HET may be useful for treating cisplatin-induced muscle atrophy.

## Data Availability

The miRNA-seq data presented in the study are publicly available. This data can be found in the GEO database, accession number: GSE295648. Other raw data supporting the conclusions of this article will be made available by the authors, without undue reservation.
