# Supplementary material for: Hochuekkito accelerates recovery from cisplatin induced-muscle atrophy accompanied by slow-twitch fiber-specific microRNA upregulation in mice
Source: Front Pharmacol. 2025 May 15;16:1502563. doi: 10.3389/fphar.2025.1502563 (PMC12141861; doi:10.3389/fphar.2025.1502563)
Supplement: Supplementary file 1 [file DataSheet1.zip › Supplymentary Materials.docx]

Supplementary Material

# Supplementary Data

Supplemental File_materials.xlsx

Supplemental File_miRNA-DESeq2.xlsx

Supplemental File_miRNA-analysis data.xlsx

# Supplementary Figures

**
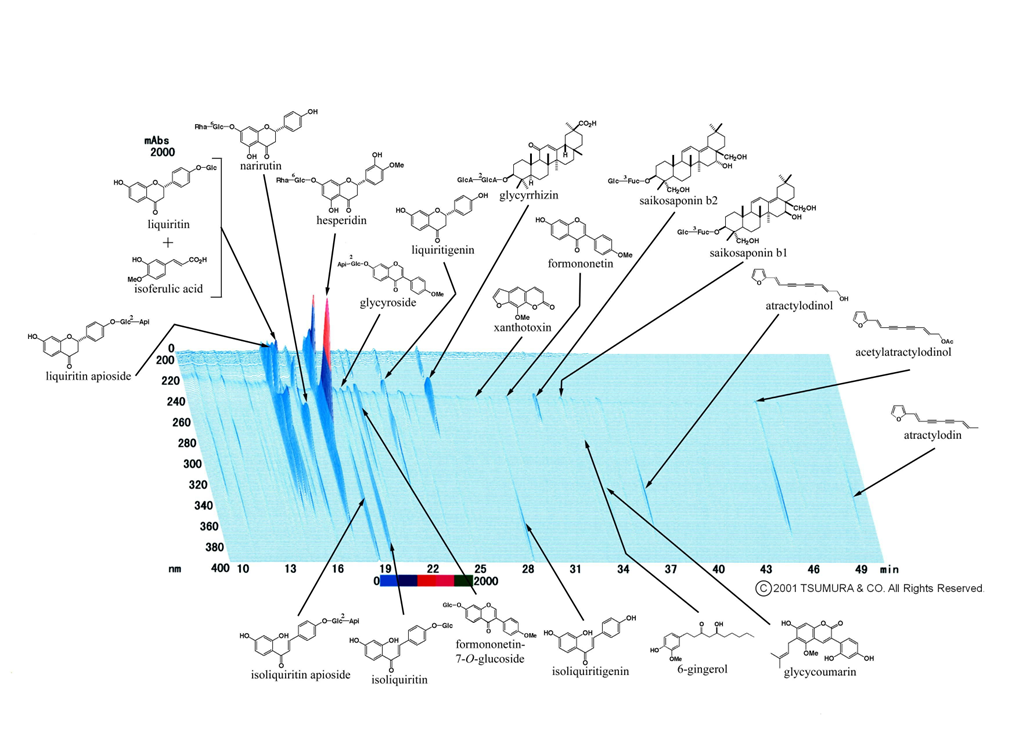
**

**Supplementary Figure 1.** A three-dimensional high-performance liquid chromatogram of hochuekkito.

**
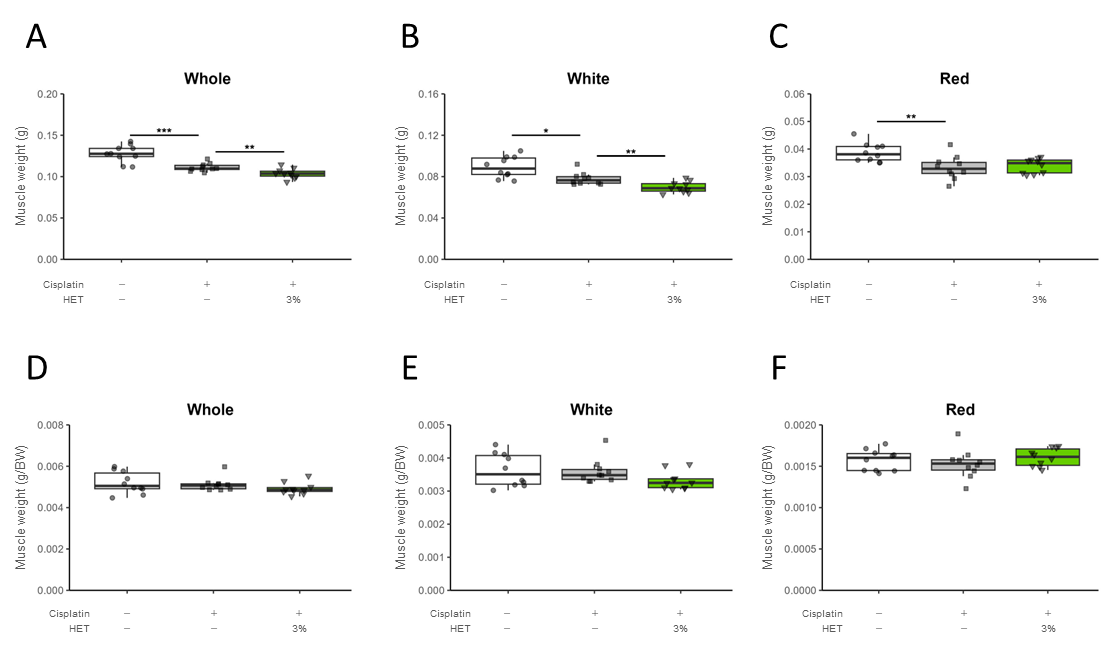
**

**Supplementary Figure 2.** The loss of muscle weight due to cisplatin treatment and the effect of HET administration on Day7. (A) Whole gastrocnemius muscle, (B) white muscle, (C) red muscle region weight, and (D-F) muscle weights per gram body weight on the day of dissection on Day7. *n* = 10 for each group. Data are presented as Tukey's boxplots. The white box and round dots, grey box and squares, and green box and downward triangles represent the vehicle, cisplatin, and 3% HET groups, respectively. The dots represent the values for each animal. * *p* < 0.05, ** *p* < 0.01, *** *p* < 0.001 by Tukey’s multiple comparison test with robust regression model.

**
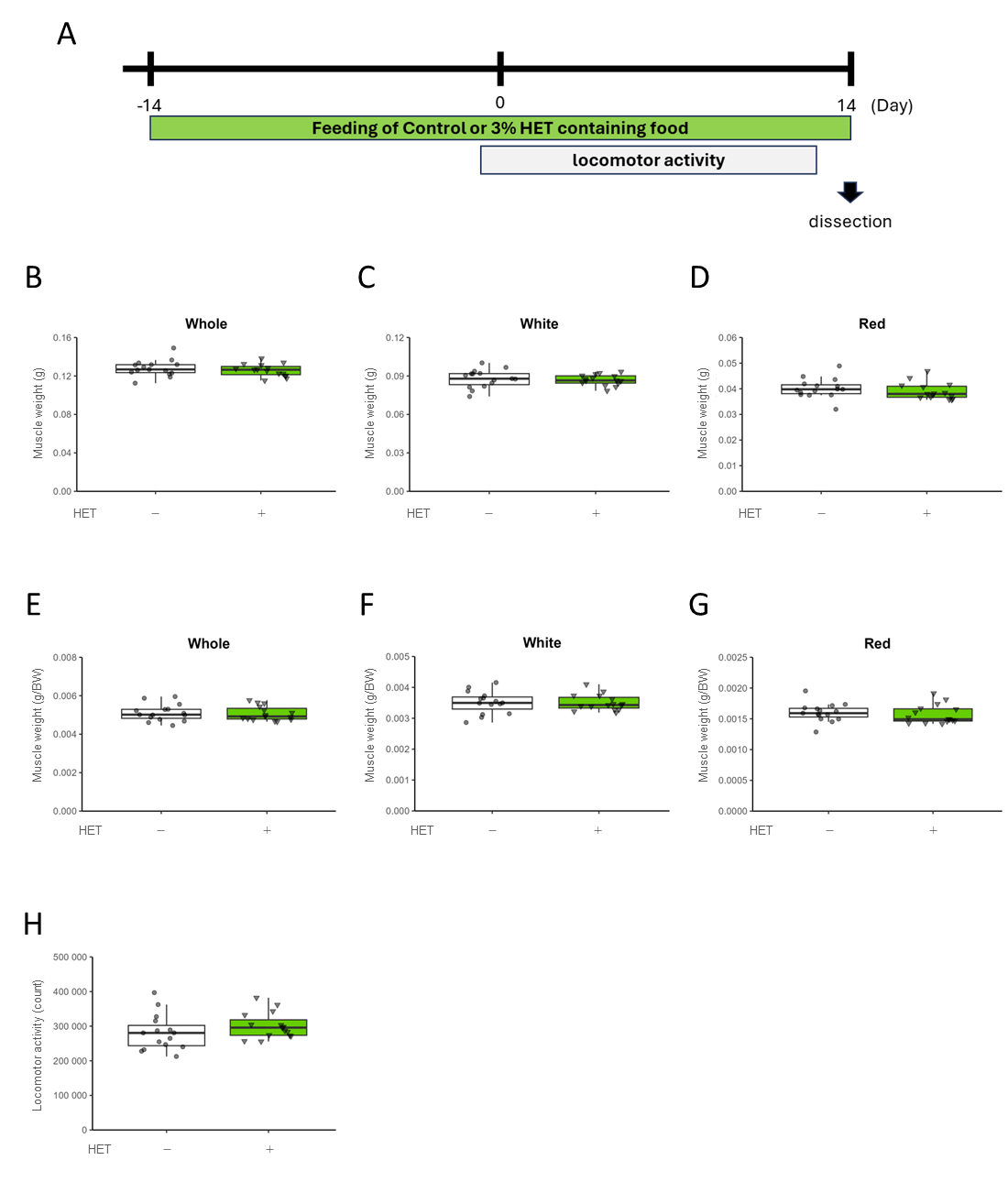
**

**Supplementary Figure 3.** Effect of HET administration in normal mice. (A) Experimental design. (B) Whole gastrocnemius muscle, (C) white muscle, (D) red muscle region weights and (D-G) muscle weights per gram body weight on the day of dissection. (H) Cumulative locomotor activity for 14 days (21 h/day). *n* = 15 for each group. Data are presented as Tukey's boxplots. The white box and round dots and green box and downward triangles represent the control and 3% HET groups, respectively. The dots represent the values for each animal. No statistically significant differences were observed between each group (Student’s or Aspin–Welch *t*-test or Wilcoxon rank-sum test).


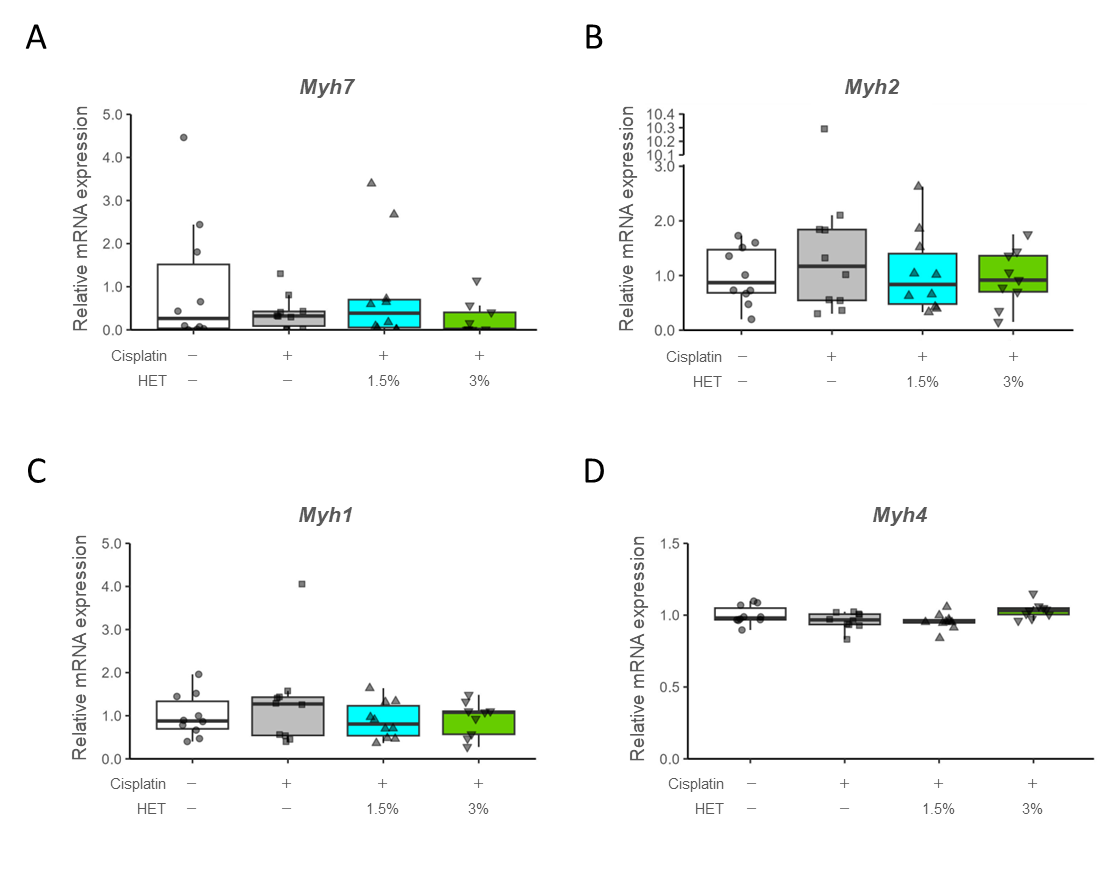


**Supplementary Figure 4.** Effects of HET administration on myosin heavy chain gene expressions in the white muscle region. (A) *Myosin heavy chain 7* (*Myh7*), (B) *Myosin heavy chain 2* (*Myh2*), (C) *Myosin heavy chain 1* (*Myh1*), and (D) *Myosin heavy chain 4* (*Myh4*) mRNA expression. *n* = 10 for the vehicle, cisplatin, and 1.5% HET groups; *n* = 9 for the 3% HET group. Data are presented as Tukey's boxplots. The white box and round dots, gray box and square dots, blue box and upward triangles, and green box and downward triangles represent the vehicle, cisplatin, 1.5% HET, and 3% HET groups, respectively. The dots represent the values for each animal. No statistically significant differences were observed between groups (Tukey’s multiple comparison test with a robust regression model).


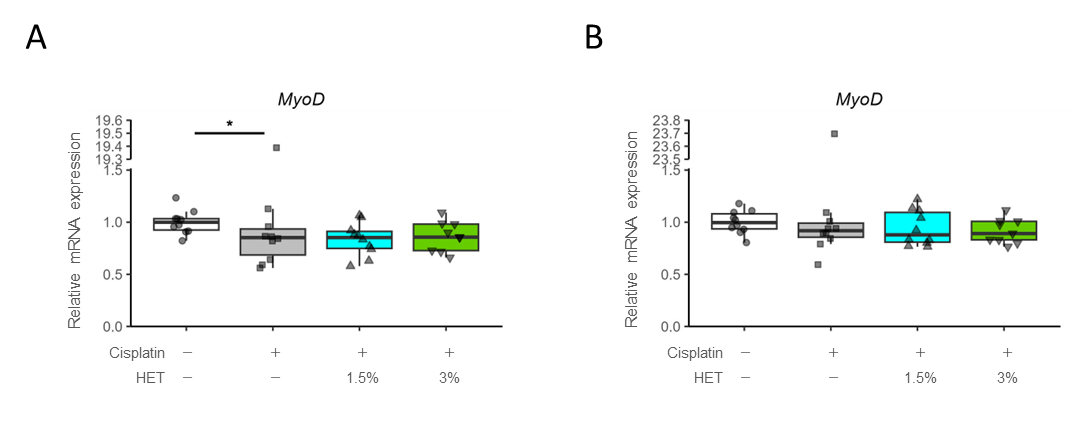


**Supplementary Figure 5.** Effect of HET on *Myogenic differentiation 1* (*MyoD*) expression in (A) white and (B) red muscle regions. *n* = 10 for the vehicle, cisplatin, and 1.5% HET groups; *n* = 9 for the 3% HET group. Data are presented as Tukey's boxplots. The white box and round dots, gray box and square dots, blue box and upward triangles, and green box and downward triangles represent the vehicle, cisplatin, 1.5% HET, and 3% HET groups, respectively. The dots represent the values for each animal. * *p* < 0.05, Tukey’s multiple comparison test with a robust regression model.


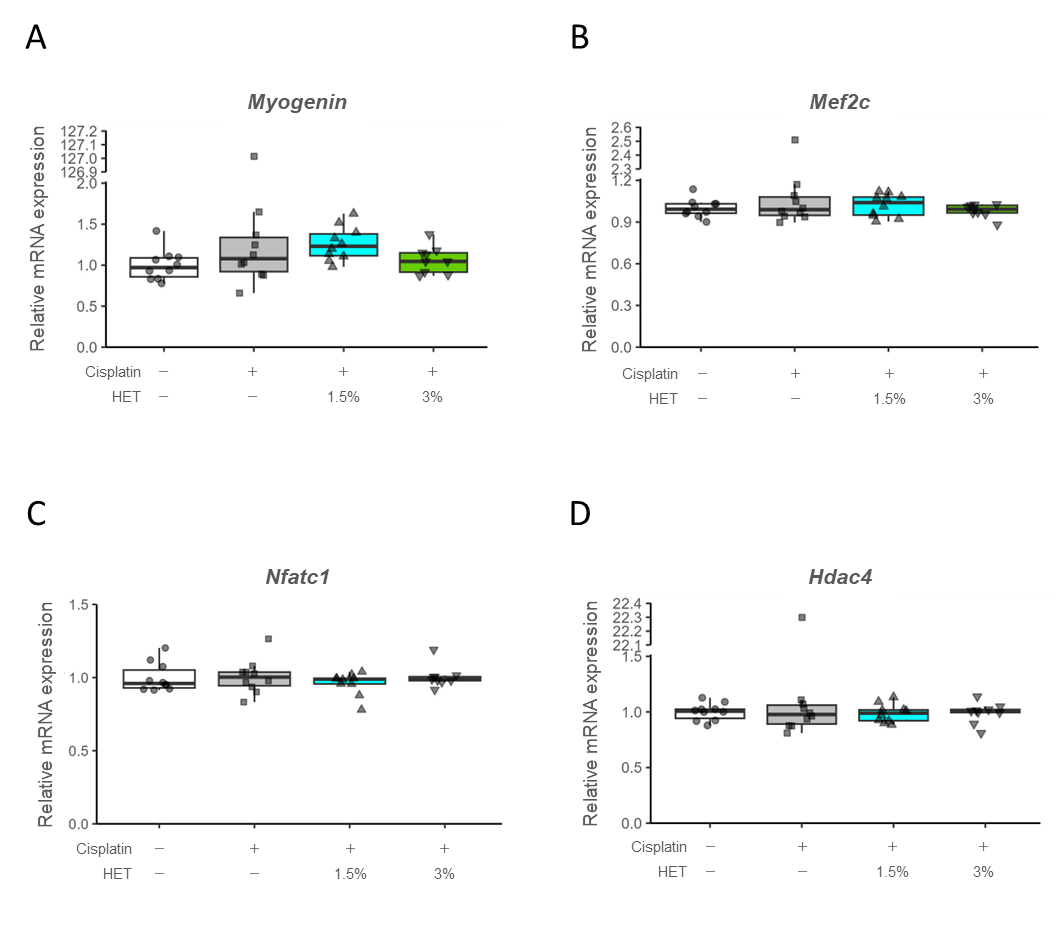


**Supplementary Figure 6.** Effects of HET on (A) *Myogenin*, (B) *Myocyte enhancer factor-2c* (*Mef2c*), and (C) *Nuclear factor of activated T cell 1* (*Nfatc1*), (D) *Class Ⅱ histone deacetylase 4* (*Hdac4*) mRNA expression in the red muscle region. *n* = 10 for the vehicle, cisplatin, and 1.5% HET groups; *n* = 9 for the 3% HET group. Data are presented as Tukey's boxplots. The white box and round dots, gray box and square dots, blue box and upward triangles, and green box and downward triangles represent the vehicle, cisplatin, 1.5% HET, and 3% HET groups, respectively. The dots represent the values for each animal. No statistically significant differences were observed between groups (Tukey’s multiple comparison test with a robust regression model).


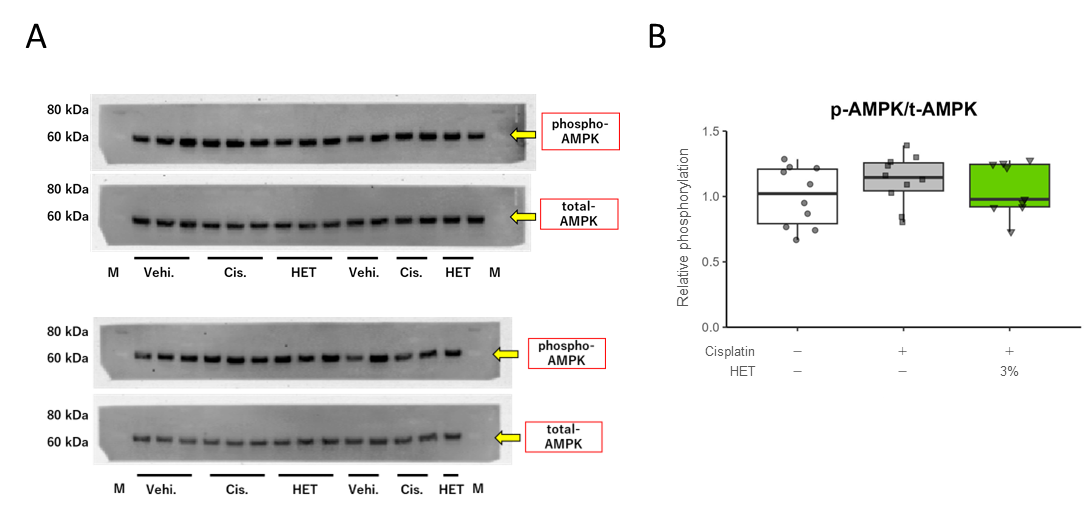


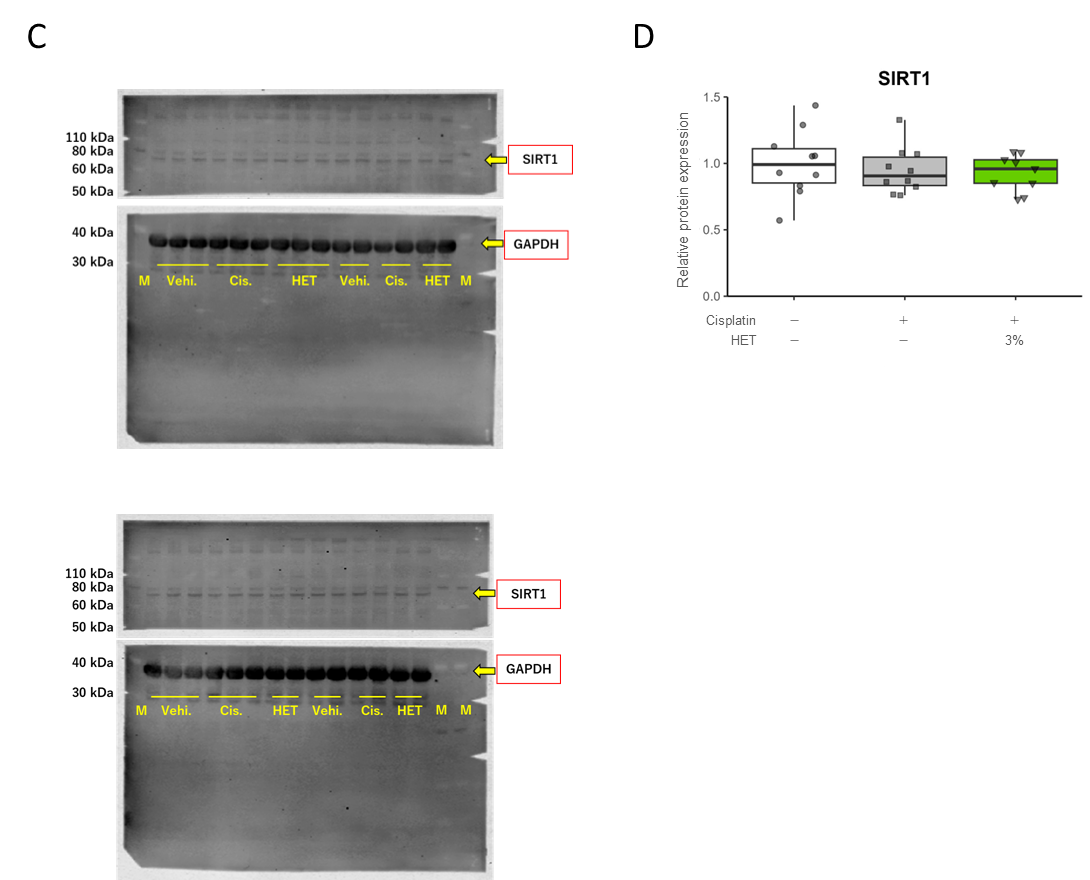


**Supplementary Figure 7.** Effects of HET on AMPK and SIRT1 protein levels on Day14 in the red muscle region. (A, B) Phosphorylated AMPK/total AMPK and (C, D) SIRT1 expression. (A, C) western blot image from two split PVDF membranes. M, standard marker; Vehi., vehicle; Cis., cisplatin; HET, 3% HET. (B, D) Quantification of protein expression based on western blot images. *n* = 10 for the vehicle and cisplatin groups; *n* = 9 for the 3% HET group. Data are presented as Tukey's boxplots. The white box and round dots, grey box and squares, and green box and downward triangles represent the vehicle, cisplatin, and 3% HET groups, respectively. The dots represent the values for each animal. No statistically significant differences were observed between the groups (Tukey–Kramer test).


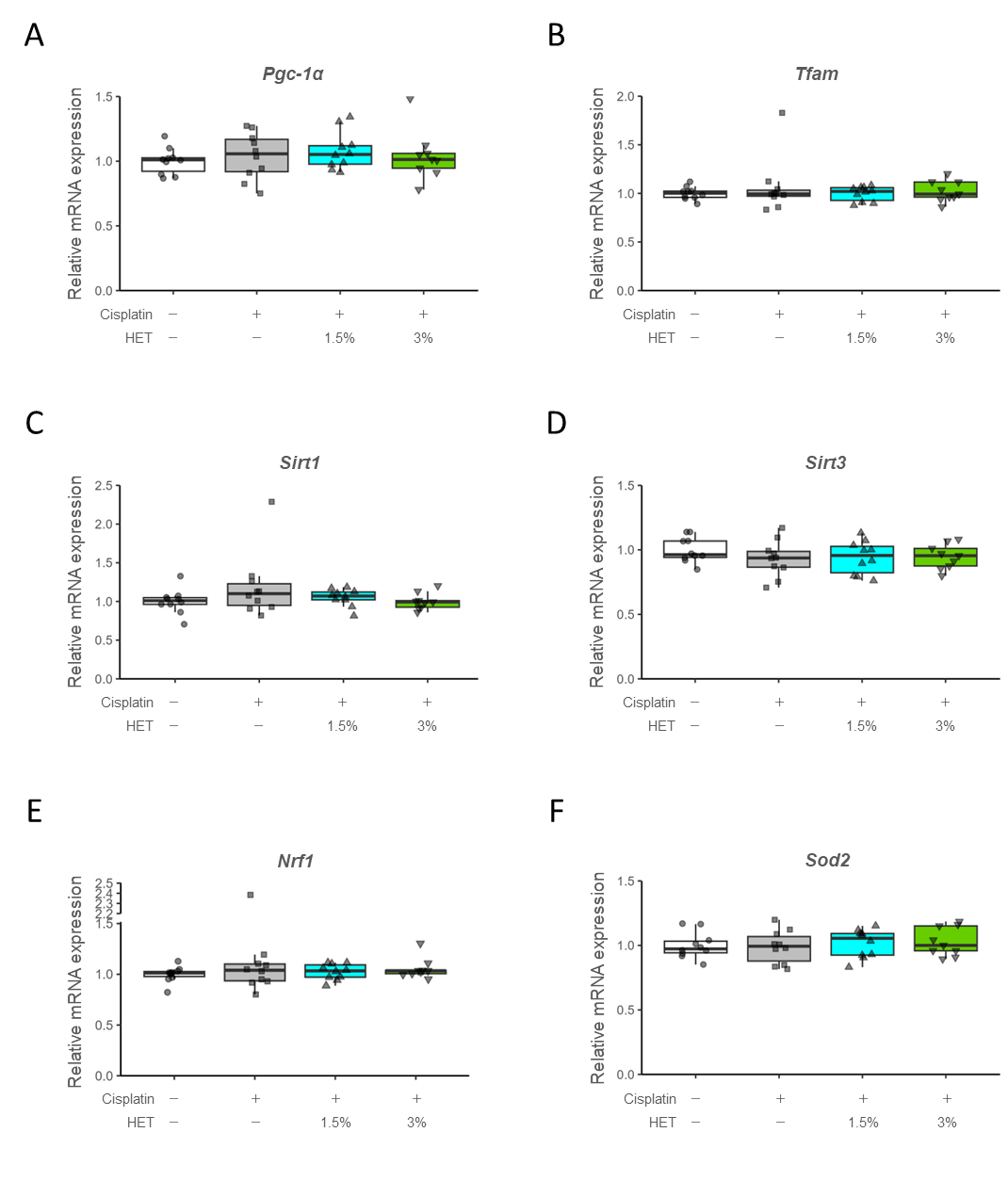


**Supplementary Figure 8.** Effects of HET on mitochondria-related gene expression on Day14 in the red muscle region. (A) *Peroxisome proliferator-activated receptor gamma coactivator 1α* (*PGC-1α*), (B) *Mitochondrial transcription factor A* (*Tfam*), (C) *Sirtuin 1* (*Sirt1*), (D) *Sirtuin 3* (*Sirt3*), (E) *Nuclear respiratory factor 1* (*Nrf1*) and (F) *Superoxide dismutase 2* (*Sod2*) mRNA expression. *n* = 10 for the vehicle, cisplatin, and 1.5% HET groups; *n* = 9 for the 3% HET group. Data are presented as Tukey's boxplots. The white box and round dots, gray box and square dots, blue box and upward triangles, and green box and downward triangles represent the vehicle, cisplatin, 1.5% HET, and 3% HET groups, respectively. The dots represent the values for each animal. No statistically significant differences were observed between the groups (Steel–Dwass or Tukey’s multiple comparison test with a robust regression model).


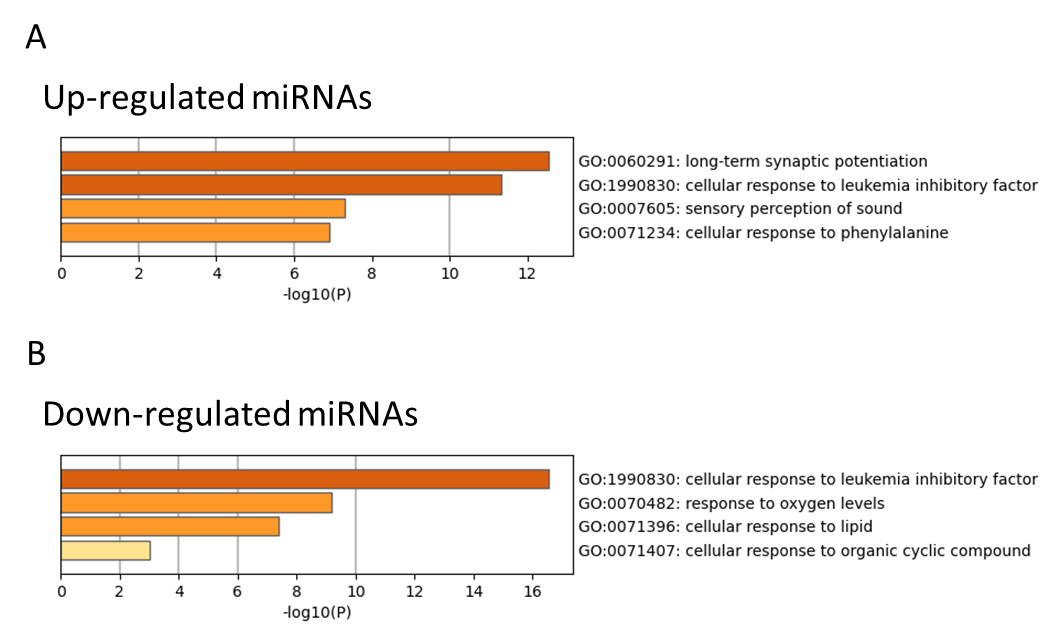


**Supplementary Figure 9.** Comprehensive analysis of miRNAs altered by cisplatin in the red muscle region. Gene Ontology analyses by Metascape were performed using either (A) the nine miRNAs upregulated or (B) nine miRNAs downregulated by cisplatin administration on Day14 (“Supplemental file_miRNA-analysis”). Terms with *p*-value < 0.01, minimum count of three, and enrichment factor > 1.5 were collected and grouped into clusters based on membership similarities. Enrichment factor: ratio of observed counts and counts expected by chance.


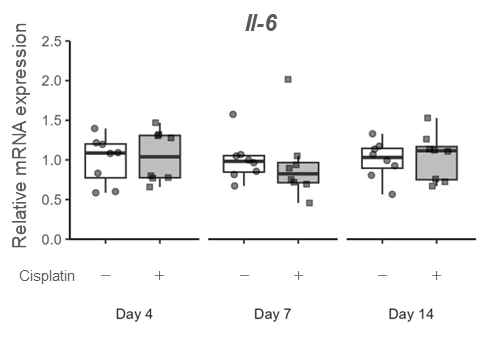


**Supplementary Figure 10.** Temporal changes in the expression of *Interleukin-6* (*Il-6*) mRNA in hind limb skeletal muscles (gastrocnemius and plantaris muscles) due to cisplatin treatment. *IL-6* mRNA levels were measured on Day4, Day7, and Day14. *n* = 8 for each group. Data are presented as Tukey's boxplots. The white box and round dots and gray box and square dots represent the vehicle and cisplatin groups, respectively. The dots represent the values for each animal. No statistically significant differences were observed between the groups (Aspin–Welch *t*-test or Wilcoxon rank-sum test).
